# Dementia in Diabetes: The Role of Hypoglycemia

**DOI:** 10.3390/ijms24129846

**Published:** 2023-06-07

**Authors:** Khaled Hameed Husain, Saud Faisal Sarhan, Haya Khaled Ali Abdulla AlKhalifa, Asal Buhasan, Abu Saleh Md Moin, Alexandra E. Butler

**Affiliations:** 1 School of Medicine, Royal College of Surgeons in Ireland, Busaiteen, Adliya 15503, Bahrain; 20200259@rcsi-mub.com (K.H.H.); 20207001@rcsi-mub.com (S.F.S.); 20200304@rcsi-mub.com (H.K.A.A.A.); 20200564@rcsi-mub.com (A.B.); 2 Research Department, Royal College of Surgeons in Ireland, Busaiteen, Adliya 15503, Bahrain; amoin@rcsi.com

**Keywords:** hypoglycemia, diabetes, dementia, Alzheimer’s disease, cognition, insulin

## Abstract

Hypoglycemia, a common consequence of diabetes treatment, is associated with severe morbidity and mortality and has become a major barrier to intensifying antidiabetic therapy. Severe hypoglycemia, defined as abnormally low blood glucose requiring the assistance of another person, is associated with seizures and comas, but even mild hypoglycemia can cause troubling symptoms such as anxiety, palpitations, and confusion. Dementia generally refers to the loss of memory, language, problem-solving, and other cognitive functions, which can interfere with daily life, and there is growing evidence that diabetes is associated with an increased risk of both vascular and non-vascular dementia. Neuroglycopenia resulting from a hypoglycemic episode in diabetic patients can lead to the degeneration of brain cells, with a resultant cognitive decline, leading to dementia. In light of new evidence, a deeper understating of the relationship between hypoglycemia and dementia can help to inform and guide preventative strategies. In this review, we discuss the epidemiology of dementia among patients with diabetes, and the emerging mechanisms thought to underlie the association between hypoglycemia and dementia. Furthermore, we discuss the risks of various pharmacological therapies, emerging therapies to combat hypoglycemia-induced dementia, as well as risk minimization strategies.

## 1. Introduction

Hypoglycemia is a known adverse effect of glucose-lowering therapies. Hypoglycemia is defined as plasma glucose levels below 70 mg/dL, as per the American Diabetes Association guidelines [1]. The manifestations of hypoglycemia vary from symptomatic stress responses to cholinergic responses and cognitive decline [2,3]. Multiple diabetic drugs can induce hypoglycemia, predominantly including, but not limited to, insulin in type 1 diabetes (T1D) and type 2 diabetes (T2D), and sulfonylureas (in T2D); there have also been associations with biguanides and thiazolidinediones [4]. With the onset of hypoglycemia, multiple counterregulatory responses act to reverse the hypoglycemic state, including a reduction in pancreatic ß-cell insulin release and increased pancreatic α-cell glucagon release through ATP-sensitive potassium (KATP) channel and glucokinase expression; a sympathoadrenal response where the hypoglycemic activation of the adrenal medulla induces epinephrine and norepinephrine secretion; and a delayed cortisol and growth hormone response [5,6].

The prevalence of hypoglycemic episodes ranges from 10% in T2D to 50% in T1D; however, despite the overall prevalence, the relationship between hypoglycemia and dementia typically refers to the elderly population as, over time, there is a reduced awareness of hypoglycemic symptoms, in addition to reduced counter-regulatory glucagon secretion [7,8]. Combined, these effects amplify the hypoglycemic risk of glucose-lowering therapies in elderly patients [9,10]. 

Dementia is increasing in global prevalence, with the current age-standardized prevalence being 5–7% in most countries [11]. Alzheimer’s dementia (AD) accounts for approximately 60% to 70% of cases, followed by vascular dementia (VD) (25%), Lewy-body dementia (LBD) (<5%), and frontotemporal dementia (FTD) (<1%). However, in diabetic patients, VD predominates followed by AD [12]. A meta-analysis of 28 prospective observational studies determined that individuals with diabetes have a 73% greater risk of developing all-cause dementia (dementia not limited to a certain type), with a 56% and 127% increased risk of AD and vascular dementia, respectively [13]. 

The mechanisms underlying the relationship between diabetes and dementia are yet to be clearly defined. The available mechanistic evidence regarding hypoglycemia secondary to diabetes mellitus, and resultant dementia, suggests multiple hypotheses based on the following: structural changes as a result of a reduced volume of grey matter and cortical atrophy; cognitive decline due to damage to the hippocampus; and a higher relative risk of neuronal damage in diabetes versus non-diabetics [2]. However, a concise molecular metabolic sequence of events addressing the connection between diabetes and dementia is still lacking in the published literature, as is the relationship of microvascular, macrovascular, and inflammatory effects on these metabolic processes that serve as a link between exposure and outcome.

There is increasing evidence that hypoglycemic episodes in patients with diabetes are a risk factor for dementia. However, as noted, the causal relationship remains relatively unclear, with multiple studies concluding that further research is necessary. Hence, a deeper understanding of the mechanistic relationship between the two events is vital, and this review article will therefore assess the published evidence reporting metabolic mechanistic determinants underlying hypoglycemia and dementia and will connect the various processes. Furthermore, this review will assess the relationship from a broader perspective, as we will explore the various emerging strategies that can help to combat hypoglycemia-induced dementia.

## 2. Hypoglycemic Events in Type 1 and Type 2 Diabetes Mellitus

A randomized controlled trial conducted on patients with T1D reported a threefold increase in severe hypoglycemia in intensively treated versus conventionally treated patients [14], with intensive therapy involving three or more daily injections of insulin as opposed to one and two insulin injections daily in the conventional therapy group. Moreover, approximately 2–4% of deaths in people with T1D are due to hypoglycemia [15]. A study was undertaken in which patients with T1D and T2D (n = 267) were randomly surveyed, recording the number of hypoglycemic events within a period of 4 weeks [16]. The results showed that 155 diabetes patients reported 572 incidents of hypoglycemia. In T1D patients, these numbers equate to a rate of ~43 hypoglycemic events per patient per year, whereas insulin-treated T2D patients experience a rate of ~16.4 events per patient per year. Self-reports of hypoglycemia in T2D patients were lower than those of T1D; however, the authors concluded that, in insulin-treated T2D patients, hypoglycemia (significant enough to cause morbidity) occurs more often than is reported [16]. Furthermore, it is more difficult to obtain data regarding T2D due to how much it varies in different regions [17]. Typically, patients with T2D diabetes are middle-aged/elderly, and therefore accurate figures for the frequency of hypoglycemia may be markedly underestimated [18].

## 3. Dementia in Patients with Diabetes

### 3.1. Association of Hyperglycemia and Dementia in Patients with Diabetes

Multiple studies have identified an association between hyperglycemia, hypoglycemia, and cognitive impairment in individuals with diabetes. The risk of dementia is suggested to increase by 25–91% in diabetic versus non-diabetic patients, with AD and VD being the more frequent subtypes [19]. The ACCORD-MIND cross-sectional study showed that a hyperglycemic increment of 1% in glycosylated hemoglobin (HbA1c) was linked to a 1.75-point lower Digital Symbol Substitution Test (DSST) score, a 0.14-point lower Mini-Mental State Examination (MMSE) score, and a more unfavorable Stroop test score (0.75s increase) [20]. These scores demonstrated impairment in psychomotor speed, memory, and executive function. The significant negative association between HbA1c level and cognitive function found in the ACCORD-MIND study has been confirmed in multiple other studies as well [19]. Impairment of cognitive function differs between individuals with T1D and T2D. Cognitive dysfunction in T1D largely affects mental flexibility, causing slowing of mental speed but not largely affecting memory and learning whereas, in T2D, psychomotor speed, executive function, and memory are largely affected [19].

Hyperglycemia in diabetes results from the insufficient action of insulin. The brain has a large number of insulin receptors, specifically in the cerebral cortex and hippocampus; these areas play major roles in cognitive function and memory, respectively [19]. The mechanism through which hyperglycemia affects cognitive function is dependent upon whether it is acute or chronic. In acute hyperglycemic cases, poor glycemic control in both T1D and T2D leads to a reduction in cerebral blood flow; the resultant osmotic changes cause oxidative stress, a self-propagating phenomenon leading to the excessive production of reactive oxygen species (ROS) [21]. The brain’s weak antioxidant capacity makes it particularly susceptible to excessive oxidation insults. When ROS production exceeds antioxidant response capacity, neuronal damage occurs with subsequent functional decline [22]. Chronic hyperglycemia also contributes to neuronal damage via the accumulation of advanced glycation end products (AGEs) [23]. In diabetes, AGE accumulation is exacerbated, causing the formation of ROS and proinflammatory cytokines, which subsequently lead to microvascular and systemic changes [24]. Moreover, it has been suggested that AGEs can accelerate Aβ deposition and plaque formation, which are pathological hallmarks of AD [25].

### 3.2. Dementia due to Diabetic Micro- and Macrovascular Complications

Reports of the association between dementia in diabetic patients due to diabetic micro- and macrovascular changes have led to diabetes being recognized as a risk factor for VD [26]. Whilst the mechanism underlying this association is not completely understood, studies show that cerebral small vessel disease (SVD) in patients with diabetes is thought to have a similar pathogenic role to diabetic microvascular disease (retinopathy and nephropathy) [19]. The homology of cerebrovascular and retinal cells, as well as their small vessel microvasculature consequent upon their similar embryological origin, provides some insight into the similarities [27]. SVD in diabetics, presenting on MRIs as silent brain infarcts, can lead to cognitive decline depending upon the region infarcted. Diabetic retinopathy has been used as a “surrogate marker” for cognitive impairment in patients with diabetes [28]. From a macrovascular perspective, diabetes is a known risk factor for both symptomatic and asymptomatic macrovascular disease, which arise as a consequence of abnormal metabolic processes in uncontrolled diabetes (increasing oxidative stress, protein kinase C activation, activation of AGE receptors) and contribute to thrombosis and hypertension [29].

### 3.3. Ambulatory Glucose Profile in Diabetes-Related Dementia

The ambulatory glucose profile (AGP) utilizes continuous glucose monitoring (CGM) systems in patients as part of their care and generates a standardized one-page summary report of their glycemic control status [30]. CGM is a device that measures blood glucose levels through a subcutaneous sensor on the upper arm or abdomen. This sensor is attached to an external transmitter, recording the blood glucose levels [31]. CGM allows diabetic individuals to track and adjust their insulin and carbohydrate needs by alerting them when their glucose levels exceed or fall below normal levels or when rapid changes occur [32]. This comprehensive overview, incorporating a statistical and visual summary of glucose profile, offers patients a better understanding of their own glycemic control and allows for improvement in the appropriate decision-making process of physicians and diabetes care teams through optimal monitoring. An AGP is obtained when information is then condensed into a “modal” or standard day as if they had been taken over a 24 h period, and reanalyzed in terms of treatment-relevant factors [33]. One important component of the AGP is time in range (TIR) reporting the proportion of time spent within the allowable range, thus allowing for the determination of time out of the target glycemic range of 70–180 mg/dl (HbA1c ~7%). Studies carried out on various patient populations with a range of CGM devices have confirmed that >70% of a patient’s day should be spent inside this range [34]. A strong correlation exists between HbA1c and %TIR, each 10% point increase in TIR producing an approximate 0.8% decrease in HbA1c, further supporting %TIR as a preferred metric in assessing the risk of diabetes complications. A reduction in %TIR has reportedly been linked with an increased risk of micro- and macrovascular complications [34], with evidence showing that a 10% reduction adds a 40% hazard risk increase for retinopathy development [35], as well as an increased risk for all-cause and cardiovascular disease-related mortality [36]. No studies were found providing a direct association between %TIR and dementia; however, regular adjustments in therapy after assessing changes in %TIR could support a reduction in diabetic-related diseases.

### 3.4. Type 2 Diabetes and Cognition in Older Adults

Older patients represent a heterogeneous group, ranging from healthy individuals to those with multiple comorbidities, making disease management and treatment in the latter group a complex and challenging task for clinicians [37].

Multiple factors influence the development of diabetes in elderly patients. Insulin resistance increases with aging; this occurs for several reasons, including changes in body composition and a decrease in physical activity. Additionally, aging is associated with impaired glucose tolerance, defined by the American Diabetes Association (ADA) as “two-hour glucose levels of 140 to 199 mg per dL (7.8 to 11.0 mmol) following a 75 g oral glucose tolerance test” [38,39]. Studies have shown that aging can impair insulin secretion from pancreatic β cells in response to endogenous incretins. This induces mitochondrial dysfunction and, ultimately, β-cell death. In some elderly patients, both insulin sensitivity and secretion are decreased, eventually leading to impaired glucose tolerance and subsequent diabetic manifestations [40]. A longitudinal study also showed that, in patients with diabetes, increased loss of brain volume and elevated HbA1c occurred with aging [41]. Elderly diabetic individuals are hence more likely to develop mild cognitive impairment (MCI), all-cause dementia, and AD, and T2D is associated with a 2.5-fold increased risk of dementia development [40]. Cognitive impairment in elderly individuals with T2D is influenced by several factors, including HbA1c, hypoglycemic events, inflammation, depression, and macro-/microvascular pathology. All these factors lead to vascular diseases, which subsequently increase the risk of cognitive impairment and neurologic disorders in the aging brain.

### 3.5. Type 2 Diabetes and Alzheimer’s Disease Dementia

Correlations between diabetes, particularly T2D, and AD have been the focus of many epidemiological studies. Both diseases share common pathological abnormalities such as insulin resistance, increased oxidative stress, impaired glucose metabolism, and deposition of amyloid proteins. Because the two diseases demonstrate disruption in common cellular and molecular pathways, they are thought to potentiate and aggravate one another [42]. The overlap in biochemical and molecular features has even caused AD to be referred to as “type 3 diabetes,” a form that specifically affects the brain [43]. The decrease in insulin signaling in T2D leads to a decrease in neuronal intracellular glucose bioavailability, reducing the production of acetylcholine precursors, which consequently downregulates cognitive synaptic transmission [44]. The majority of insulin receptors are localized at the entorhinal cortex, hippocampus, and frontal lobe regions, and significant hippocampal and amygdalar atrophy has been demonstrated on magnetic resonance imaging (MRI) in T2D, regardless of vascular pathology [45].

Insulin and Aβ share a common degradation pathway implemented by the insulin-degrading enzyme (IDE), consequently leading to competition at elevated concentrations. IDE not only degrades insulin and amylin but also T2D-related peptides and Aβ peptides in the AD brain. Competition during hyperinsulinemia may lead to the elevation of Aβ via the competitive inhibitory effect of insulin on IDE. This has led to the suggestion that IDE is the link between AD and hyperinsulinemia. Furthermore, given that IDE is more insulin-selective than Aβ, brain hyperinsulinemia may impact the clearance of Aβ, favoring its cerebral accumulation and its consequent neuroaccumulation [46].

Furthermore, hyperglycemia and hyperinsulinemia induce tau hyperphosphorylation and amyloid oligomerization, thereby accelerating brain aging by promoting dispersed microangiopathy in the brain. Insulin resistance has also been shown to alter the intracellular signaling cascade of MAPK (mitogen-activated protein kinase), GSK-3 (glycogen synthase kinase-3), and phosphoinositide 3-kinase (PI3K). All these pathways lead to increased tau hyperphosphorylation. Moreover, the reduced expression of GLUT-1 and GLUT-3 glucose transporters in different regions of the brain has been shown to cause the downregulation of hexosamines, which consequently reduces O-GlcNAcylation and promotes tau hyperphosphorylation as well [44].

### 3.6. The Role of Cerebral Glymphatic System Dysfunction in Diabetes Mellitus

The clearance of excess fluid and toxins from the brain is essential for maintaining homeostasis. This homeostasis is achieved by the cerebral glymphatic system (CGS). This system follows a three-step process, starting with cerebrospinal fluid (CSF) transportation from basal cisterns into the subarachnoid space covering the cerebral hemispheres. Then, driven by bulk flow, the CSF enters the periarterial spaces. Secondly, aquaporin 4 water channels (AQP4), found on astroglial end-feet, allow cerebrospinal–interstitial fluid (CSF–ISF) and waste solute removal to mix together by propelling the CSF from the periarterial compartment into the ISF. Lastly, this fluid mixture moves to the perivenous compartment of the larger central veins and then exits into the left ventricles and eventually into the circulatory system [47].

CGS dysfunction can be attributed to several causes, including diabetes and aging [48]. Diabetes causes the suppression of interstitial fluid clearance in both the hippocampus and hypothalamus, hence contributing to CGS dysfunction [49]. Additionally, with aging, several changes occur to the glymphatic system, including a reduction in cerebrovascular pulsatility, a decrease in AQP expression, and its mislocalization from the astroglia end-feet [48,50]. This leads to a 40% decrease in cerebral Aβ clearance. The inability of the CGS to completely clear tau proteins and Aβ accumulations leads to their build-up in the interstitial space and ultimately results in cognitive decline during aging and AD [50]. Patients with AD are likely to develop worsening glymphatic clearance, a system that reduces the deposition of Aβ [50].

### 3.7. Neurodegeneration in Obese Patients with Type 2 Diabetes

As with T2D, the risk of neurodegeneration is also increased in obese patients. Obesity prevalence worldwide has increased significantly and is expected to increase further in subsequent decades (573 million by the year 2030) [51].

A major cause of obesity is the consumption of a high-fat diet. Chronically elevated levels of fatty acids cause lipid accumulation in adipocytes. This has several negative effects, including low-grade inflammation [51,52]. Obesity is thus a major risk factor for insulin resistance and related diseases such as metabolic syndrome and T2D. By increasing the expression of inflammatory cytokines, signaling pathways that interfere with insulin action and signaling are activated [52].

Obesity, independent of T2D, is associated with dementia. A high-fat diet activates the immune system, thereby promoting AD pathogenesis. Saturated fatty acids cause an inflammatory response through Toll-like receptor 4 (TLR4) in the hypothalamus. TLR4 protein detects lipopolysaccharides and, when activated, causes cytokine generation in astrocytes. Studies have shown that the risk of developing dementia is three times greater in those with a larger waist diameter than those with a smaller one. A larger waist–hip ratio has also been found to be associated with hippocampal volume decrease. Moreover, a study conducted by Xu et al. reported that mid-life obese individuals developed dementia at a mean odds ratio (OR) of 3.88, again suggesting that obesity in mid-life is associated with dementia [53].

## 4. Dementia Due to Hypoglycemia 

### 4.1. Epidemiology of Hypoglycemia, Cognition, and Dementia among Diabetics

Hypoglycemia is very common among patients with T1D and T2D. Alwafi et al. performed a systematic review encompassing 2,462,810 individuals and spanning all continents and showed that the prevalence of hypoglycemia among diabetics ranged from 0.074% to 73.0%, with the highest incidence and prevalence observed in T1D patients and those treated with insulin (prevalence range of 2.2% to 73.0%); additionally, the pooled prevalence among European and North American self-reported, cross-sectional studies included in this meta-analysis were 35.0% (95% CI, 32.0–38.0, I^2^ = 59%) and 11.0% (95% CI, 11.0–13.0, I^2^ = 38%), respectively [54]. Studies assessing cognitive status among diabetics have also suggested this as a useful metric. A study concluded that, among diabetics, 63% of the study population have reduced cognition using the Mini-Mental State Examination (MMSE), whilst 70% were reported as having reduced cognition when assessed by the modified MMSE (3MS) [55]. Higher figures were reported in Saudi Arabia, where 80% of diabetic study subjects were noted as having a form of cognitive impairment, 33.8% of which had severe impairments [56]. With regard to dementia specifically, a UK database study showed that the incidence of dementia among diabetics increased 3.7-fold from 2000 to 2016, increasing from 0.2 cases per 100 persons to 0.7 cases per 100 persons. The prevalence of diabetic females with dementia was higher than that of diabetic men (3.1% versus 2.0%), and diabetics aged 65 and over had a substantially higher prevalence of dementia than diabetics aged 18 to 65 years of age (4.2 per 100 persons versus 0.2 per 100 persons). The study concluded that the incidence and prevalence of dementia among diabetics are increasing [57]. It is therefore apparent that the prevalence and incidence of hypoglycemia and cognitive impairment among diabetics are on the rise.

### 4.2. Association between Hypoglycemia and Dementia

Huang et al. conducted a systematic review of 10 cohort studies that encompassed Western and Asian populations, aiming to identify the risk of developing dementia secondary to hypoglycemic episodes (1 episode or >1 episode) in both T1D and T2D, with the controls being diabetics with no experience of hypoglycemic episodes [2]. This meta-analysis showed a hazard ratio (HR) of 1.44 (95% CI: 1.26, 1.65 I^2^ = 89% *p* < 0.00001) for developing dementia as a result of single or multiple severe hypoglycemic episodes requiring hospital admission (only one study included any form of hypoglycemia and was not exclusive to severe hypoglycemia). An increased risk of dementia development was observed in studies including only T2D as well as both T1D and T2D. Subgroup analyses yielded a higher OR for diabetics who experience two or more hypoglycemic episodes (HR = 1.63, I^2^ = 84% *p* = 0.02) than for those with one hypoglycemic episode (1.21 95% CI: 1.11, 1.32 I^2^ = 0% *p* < 0.0001). However, a limitation of this review was that dementia and hypoglycemia were not predefined by the authors, and hence any definition was accepted from the included studies [2]. 

Another meta-analysis conducted by Mattihsent et al. on American, European, and Asian populations involved the analysis of 44 studies (N = 2,507,434) and revealed an association of hypoglycemia with dementia, with a pooled OR of 1.50 (95% CI 1.29–1.74). Taken together, the available literature on the association of hypoglycemia and dementia suggests a significant correlation amongst multiple population groups. Table 1 lists other relevant systematic reviews noting the risk of developing dementia due to hypoglycemia.

### 4.3. Recurrent Hypoglycemia and Dementia

Many studies substantiate the claim that there is a positive correlation between the number of hypoglycemic episodes and the risk of dementia development. For example, as shown in Table 1, a meta-analysis study of T2D patients showed an increasing trend of statistically significant risk ratios as the number of hypoglycemic events increased [59]. Additionally, a population-based cohort study conducted in South Korea following 5966 patients who had at least one hypoglycemic episode further corroborated this positive correlation: The HR results were 1.170 (95% CI, 1.043–1.313), 1.201 (95% CI, 1.016–1.421), and 1.358 (95% CI, 1.060–1.740) for 1 hypoglycemic episode, 2–3 hypoglycemic episodes, and >3 hypoglycemic episodes, respectively [61]. Furthermore, another longitudinal cohort study conducted in California including 16,667 patients with T2D reported similar results. Patients were followed for 12 years (from 1990 to 2002), and hypoglycemic events were recorded and then followed for a further 5 years (until 2007) to screen for dementia. The adjusted HR for 1 hypoglycemic episode was 1.26 (1.10–1.49), for 2 hypoglycemic episodes was 1.80 (1.37–2.36), and for ≥3 hypoglycemic episodes was 1.94 (1.42–2.64) [62]. On a larger scale, a study conducted on 53,055 patients with T2D revealed a 26% increased risk of dementia development in those with one hypoglycemic episode, and a 50% increased risk in those with two or more hypoglycemic episodes [58]. Collectively, these studies suggest that patients with repeated hypoglycemic episodes have an increased risk of dementia development; however, the multifactorial nature of dementia limits the direct application of these results to daily living, as other confounding risk factors and comorbidities are likely to be present.

### 4.4. Glycemic Control and Dementia

HbA1c is widely used to determine glycemic control over the previous 3-month time span. Hence, the majority of studies use HbA1c when assessing glycemic control and dementia risk, with tight glycemic control referring to HbA1c levels below 7.0% (53 mmol/mol). Many studies identified an association between increasing HbA1c levels and increased risk of dementia. A large UK cohort study including 372,287 patients with both T1D and T2D reported an HR of 1.08 (1.07, 1.09) of developing dementia for every 1% increase in HbA1c. If tight glycemic control can be attained (HbA1c level below 6%), the HR for dementia development drops to 0.86 (0.83–0.89) [63]. A meta-analysis by Tang et al. also showed that tight glycemic control can slow cognition decline, especially in terms of memory [64]. 

### 4.5. Risk Factors of Hypoglycemia-Induced Alzheimer’s Dementia in Diabetes Mellitus

It is important to identify the risk factors of hypoglycemia-induced dementia in diabetics to allow for early intervention. Many therapeutic glucose-lowering agents can induce hypoglycemia. Such drugs include insulin and sulphonylureas, as well as co-administered medications such as beta-blockers, fluoroquinolones, and ACE inhibitors [65]. A retrospective survey conducted between 1995 and 1996 of 24,793 medical admissions in teaching hospitals showed that 0.5% of patients were hospitalized due to hypoglycemia, with 55% of these admissions due to sulphonylureas. Patients treated with insulin are particularly vulnerable to hypoglycemia, especially those who use vials and syringes rather than disposable pens [66,67]. 

In addition to drug-induced risks, in a longitudinal cohort study conducted in northern California, for which 16,667 patients enrolled, 11% of patients had a diagnosis of dementia. Among these patients, those who had hypoglycemic events were more likely to be of advanced age, African American, treated with insulin, or hypertensive, and to have had a previous stroke, or have end-stage renal disease than patients without hypoglycemia [62]. Other risk factors include prolonged fasting (during Ramadan, for example), concurrent infections, cardiovascular disease, and renal insufficiency [65,68]. Patients with renal insufficiencies, such as chronic kidney disease (CKD), often present with impaired gluconeogenesis (as approximately 20% of plasma glucose is produced by renal gluconeogenesis), and altered renal drug metabolism puts them at risk for hypoglycemia. Furthermore, renal insufficiency and hypoglycemia in T2D have been reported to have independent effects on all-cause mortality, highlighting the multiple risk factors in patients with comorbid conditions [69,70]

### 4.6. Hypoglycemia and Vascular Dementia: Are They Connected?

As mentioned in a bioinformatic study by Saik and Klimontov, there appears to be a genetic link between hypoglycemia and cardiovascular disease/diabetic microvascular complications [71]. This suggests that there may consequently be a connection between hypoglycemia and VD as well. There are limited studies assessing the pathophysiology of this purported relationship, but a study on a rat model revealed that severe hypoglycemia can cause the leakage of the blood–brain barrier and consequent brain edema. Using Evans blue extravasation into the brain as a read-out method, this experimental group observed significantly increased Evans blue content in the brain versus controls, implying that severe hypoglycemic events aggravate brain edema in diabetic mice models [72]. Furthermore, other animal and human studies demonstrated that severe hypoglycemic events with coma can also cause selective neuronal cell death in susceptible areas of the brain, particularly the hippocampus and cortex, as evidenced by magnetic resonance imaging [73].

### 4.7. Is Pre-Existing Dementia a Risk Factor for Hypoglycemia in Type 2 Diabetes?

There is scarce literature on whether pre-existing dementia acts as a risk factor for hypoglycemia in T2D. A 2015 meta-analysis screened 1175 citations, from which 10 studies (including 535,317 participants) were prioritized to include geographical diversity in patients with T2D who were receiving insulin and/or oral agents. The results suggested a reciprocal link between hypoglycemia and cognitive impairment/dementia in older patients with diabetes [74]. Patients who already had cognitive deterioration had a considerably higher chance of developing hypoglycemia, with a pooled OR of 1.61 (1.25, 2.06) [75]. In order to prevent additional cognitive deterioration in elderly patients with pre-existing dementia, less stringent blood glucose targets should be employed, coupled with the strict monitoring of hypoglycemic events.

## 5. Mechanisms of Hypoglycemia Induced Dementia in Patients with Type 2 Diabetes

### 5.1. The Role of Insulin in the Brain (Learning Memory)

Insulin, predominantly insulin receptor substrate 2 (IRS2) and its receptor, play a crucial role in learning, and their respective deficits disrupt memory. IRS1 on the other hand is mainly involved in muscle and adipose tissue [76]. Insulin is critical in the development of the nervous system, as it stimulates neurite proliferation and growth. It stimulates brain growth via its receptor, with increased expression within neurons and surrounding glial cells. Additionally, insulin plays a crucial role in the brain, as it prevents damage induced by oxidative stress possibly through its effects of increased uptake of GABA and glutamate under oxidized stress, increased glucose uptake and metabolism, and increased and decreased intracellular and extracellular adenosine, which will lead to increased uric acid formation in cortical neurons [77,78]. Insulin also inhibits apoptosis, possibly through the activation of PI3K [79]. Furthermore, insulin can also minimize the effects of ischemia, as it can induce nitric oxide (NO) production and increased NO synthase (NOS) expression, and hence improves cerebral perfusion to ischemic areas [80]. Insulin was also found to directly inhibit platelet aggregation through the NO–guanylate cyclase–cGMP pathway [81]. This antiplatelet property of insulin also demonstrates an anti-inflammatory effect, as it can reduce CD40 ligand (CD40L) release from the α-granules of platelets—a significant mediator of inflammation [81]. Furthermore, insulin minimizes Aβ toxicity by acting as a neuroprotector, where insulin inhibits the fibril formation of Aβ in a cell-free environment and on the surface of human brain pericytes, which highlights how insulin may serve to prevent the onset and progression of dementia [82]. The protective actions of insulin are diminished in cases of impaired insulin signaling, consequently increasing the chance of neurocognitive and neuropsychiatric disorders and linking cognitive dysfunction to brain insulin resistance [83].

Insulin and its receptor take part in the regulation of memory and learning through the activation of certain signaling pathways. The intracellular molecules Ras/Ra, MEK/MAP kinases, Shc, and Crb-r/SOS are involved in a signaling pathway involving long-term memory [84]. A reduction in brain insulin and insulin receptor (IR) may be associated with disorders involving memory impairment, such as AD. A study that examined the brains of AD patients with and without APOε4 homozygosity highlighted that IR density was substantially reduced in patients with AD compared with healthy age-matched individuals. Additionally, a lower CSF insulin, a higher plasma insulin, and an overall lower CSF-to-plasma insulin ratio were found in the group with AD versus the healthy controls [85]. Interestingly, enhanced memory and performance were observed in AD patients when insulin was administered, and increased plasma insulin (whilst maintaining the fasting plasma glucose baseline level constant) caused memory improvement [85].

It is important to note that all brain cells contain IR; however, it is important to acknowledge the cellular heterogeneity of the brain and that the expression of IR varies between brain cell types. Important regions to note that have a high expression of IR include the hippocampus, the hypothalamus, the cerebral cortex, the cerebellum, and the striatum. It is therefore reasonable to suggest that insulin has crucial and various functions in different regions of the brain [86,87].

### 5.2. How Chronic Hyperinsulinemia Affects Cognition

**Figure 1:**
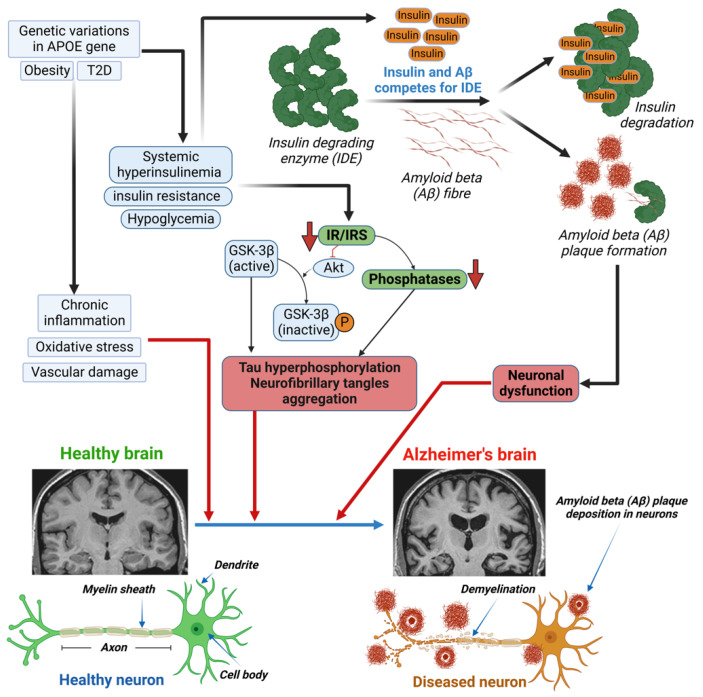
Mechanism of altered insulin levels/signaling in developing Alzheimer’s diseases (AD) in diabetes.Genetic variants in the APOE gene, obesity, or diabetes lead to chronic inflammation, oxidative stress, and vascular damage in patients with type 1 or type 2 diabetes (T1D/T2D), which can cause damage to the brain. Excess circulating insulin, due to diabetes-induced systemic hyperinsulinemia, insulin resistance, and/or hypoglycemia, causes both Aβ plaque formation and tau hyperphosphorylation. The insulin-degrading enzyme (IDE) is required for insulin and/or Aβ degradation in neurons and microglia. Elevated insulin levels induce Aβ plaque formation through competition between insulin and Aβ for the IDE. A decrease in insulin receptor (IR) or insulin receptor substrate (IRS) leads to the inhibition of Akt and dephosphorylation of GSK-3β. Decreased IR/IRS also leads to a decrease in phosphatase levels. Dephosphorylated GSK-3β is the active form that causes the hyperphosphorylation of tau protein and the aggregation of neurofibrillary tangles (NFT). Both Aβ plaque and tau hyperphosphorylation are responsible for neuronal dysfunction and the development of Alzheimer’s disease.

Hyperinsulinemia can indirectly affect cognition through vascular mechanisms. This is due to its association with micro- and macrovascular consequences in the brain, which are both involved in cognitive decline and VD development. An observational study revealed that increased mean insulin levels over a 6-year period were associated with a more substantial cognitive decline, as measured by executive function testing [88]. The mechanism of the hyperinsulinemia-induced development of AD involves the insulin-degrading enzyme (IDE) and the inhibition of Akt (Figure 1), which leads to both Aβ plaque formation and tau hyperphosphorylation, respectively [89]. The insulin-degrading enzyme (IDE) is required for insulin Aβ degradation, in both neurons and microglia. Elevated insulin levels induce Aβ plaque formation through competition between insulin and Aβ for the IDE. A decrease in IR or insulin receptor substrate (IRS) leads to the inhibition of Akt and dephosphorylation of GSK-3β. Dephosphorylated GSK-3β is the active form that causes the hyperphosphorylation of tau protein and the aggregation of neurofibrillary tangles [89].

### 5.3. Insulin Resistance and Hippocampal Dysfunction

IRs that are expressed in the hippocampus serve to regulate cognitive function. Hippocampal neuroplasticity and cognitive dysfunction are observed in metabolic disorders that include insulin resistance. A study conducted by Biessels et al. highlighted how the administration of streptozotocin (STZ), a ß-cell-ablating compound that thus induces diabetes, can lead to memory and learning impairment in rats, as determined by impaired performance in the water maze task. However, the administration of insulin to these STZ-treated rats was found to prevent impairment in memory loss and to preserve synaptic plasticity [90]. Another study demonstrated that STZ-treated rats suffered memory deficits in the hole board task, as well as the passive avoidance paradigm [91]. Moreover, correlative studies highlighted that IR is involved in memory formation and learning. Upon training rats in the spatial maze learning task, IR mRNA in the CA1 subregion of the hippocampus and the hippocampal dentate gyrus were upregulated. Moreover, the immunohistochemistry of the hippocampus showed that there was an increased expression of IR in a subpopulation of pyramidal neurons [92].

It is important to investigate the causes and consequences of hippocampal insulin resistance. It has been suggested that an increase in proinflammatory cytokines, oxidative stress, and dysfunction in the hypothalamic–pituitary–adrenal axis can cause peripheral insulin resistance [93]. Additionally, another factor that can contribute to hippocampal insulin resistance is the administration of stress levels of glucocorticoids [94].

Chronic psychological stress has also been implicated in the development of insulin resistance, leading to the development of T2D and, subsequently, diabetes-related cognitive decline. This contrasts with acute psychological stress, which has not been linked to T2D development [95]. However, the effects of chronic stress on the molecular and physiological processes involved in cognitive decline have not been clarified [96]. Chronic stress was seen to increase the deposition of Aß in a study on chronic psychological stress-induced rat models, possibly via the inhibition of amyloid precursor protein (APP), which subsequently promoted Aß generation [96]. This process was found to be inhibited when the same rat models were treated with the ZiBuPiYin recipe, a Chinese medicine formula, where the deposition of both APP and Aß was found to be reduced, making this a potential therapeutic application in diabetes-related cognitive decline induced by chronic psychological stress. Moreover, D-pinitol (DPIN), a naturally occurring inositol capable of activating the insulin pathway in peripheral tissue, has also been reported to improve insulin-resistance-associated disorders in the brain. D-pinitol lowered tau phosphorylation by regulating cyclin-dependent kinase 5 (CDK5) activity in mouse brain [97] and activated the insulin signaling pathway by increasing the phosphorylation of PI3K/Akt in rat hypothalamus [98], suggesting D-pinitol as a potential drug for the treatment of neurological disorders such as dementia.

### 5.4. The Role of Oxidative Stress and Mitochondrial Dysfunction Secondary to Hypoglycemia

In neurodegenerative disorders, ROS has been shown to cause cellular injury. ROS causes the accumulation of Aß protein in AD, thereby causing lysosomal membrane degeneration and neuronal death. Another cause for an increase in ROS production is a deficiency of cytochrome c oxidase, an enzyme in the mitochondrial electron transport chain (ETC). An increase in ROS can impede energy metabolism and decrease energy stores. Moreover, ROS also causes tau hyperphosphorylation in AD, as it inhibits phosphatase 2A, causing glycogen synthase activation (which is involved in tau phosphorylation), and the hyperphosphorylation of tau leads to neurofibrillary lesions in AD [99].

Mitochondrial dysfunction has been purported to be the predominant cause of ROS production in AD, as it results in the abnormal processing of ROS. Oxidative stress is known to cause mitochondrial dysfunction by contributing to multiple mitochondrial DNA mutations, altering Ca^2+^ homeostasis, which increases mitochondrial calcium and subsequently disturbs neuronal function, as well as attacking complex sites in the transport chain, importantly Complex I and Complex III, which can increase superoxide formation [100,101]. Moreover, altered bioenergetics can result from increased mitochondrial fission; this is thought to be due to S-nitrosylation-dynamin-related protein 1 accumulation, as postmortems of AD patients revealed an increase in this protein [102]. In familial cases of AD, it is also known that PSEN1 and PSEN2 genes are associated with altered calcium signaling in the mitochondria, which can lead to subsequent dysfunction [103]. Another factor that can lead to the generation of ROS is the insertion of Aß oligomers into the lipid bilayer of hippocampal neurons [99].

Both oxidative stress and abnormal mitotic signaling provoke AD onset, as these stressors can independently initiate cellular dysfunction; however, both are needed to initiate disease pathogenesis in neurons that cause AD, highlighting the fact that oxidative stress is a critical underlying factor [104].

### 5.5. Genetic Factors 

The APOε4 is known to be the greatest risk factor for AD and is also linked to T2D. Numerous neuropathological studies have highlighted that the highest number of AD lesions in brain tissue were found in APOε4 diabetic patients. However, it still remains unclear how the APOE isoforms affect the development of T2D and AD. Lower insulin levels in CSF can be due to prolonged hyperinsulinemia. However, this relationship is not as strong in patients who are APOε4 carriers. Additionally, patients with APOε4 are less likely to respond to insulin-related interventions [105].

Patients with cognitive impairment have been reported to have higher levels of fasting insulin. Moreover, APOε4 status can influence glucose tolerance. However, it remains unclear whether APOε4 allele modification is due to hyperinsulinemia, and hence deeper insight is needed to understand the pathogenesis of AD and how the APOE genotype can influence insulin metabolism [105].

### 5.6. Role of Coagulation Defects and Endothelial Abnormalities in the Brain

The calcium-mediated intracellular signaling pathway is one of the many mechanisms wherein thrombin causes oxidative stress in diabetes. This pathway regulates the transcription factor, KLF14, and the PLK1 kinase pathway, which leads to the increased production of ROS [106]. A mouse model of diabetes highlighted how exogenous thrombin administration leads to increased blood–brain barrier permeability, the expression of inflammatory cytokines, and a decreased expression of tight junction proteins [107]. Additionally, STZ-induced diabetes in mice increased the expression of protease-activated receptors (PARs) in the aorta. PARs are integral for the regulation of cell adhesion, inflammation, cell migration, and platelet activation [108]. These STZ-induced diabetic mice showed endothelial impairment. However, dabigatran etexilate, a direct thrombin inhibitor, remarkably improves this endothelial dysfunction [109]. 

**Figure 2:**
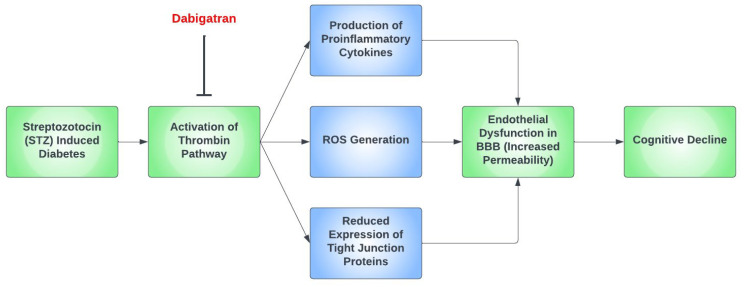
A proposed simplified pathway for cognitive decline in the setting of streptozotocin (STZ)-induced diabetic mouse models. In mouse models that had streptozotocin (STZ)-induced diabetes, the thrombin pathway was found to be activated, which ultimately led to cognitive decline through the dysfunction of the blood–brain barrier (BBB). This occurs predominantly due to the formation of proinflammatory cytokines and the generation of reactive oxygen species (ROS), as well as the reduced expression of tight junction proteins. Dabigatran has been repeatedly found to prevent this by inhibiting the thrombin pathway.

Thrombin has been proposed as a pathological mediator in AD, as thrombin [110], thrombin receptors, and PAR-1 are all elevated in AD [111]. Moreover, thrombin is present in the key pathological hallmark lesions of AD, such as neurofibrillary tangles and senile plaques. Thrombin-related pathways are also found in diabetic retinopathy and microvascular injury. A study reported increased expression of thrombin and PAR-1 in diabetic retinopathy patients [112]. Furthermore, thrombin takes part in multiple mechanisms of AD such as hippocampal degeneration, promotion of tau aggregation, and the secretion of amyloid precursor protein in endothelial cells [76]. The depletion of factor XI was found to diminish cognitive impairment in AD mice due to Aß-induced thrombin activation via factor-XII-mediated factor XI activation [113]. Figure 2 demonstrates a possible simplified pathway for cognitive decline in hyperglycemia induced by STZ in mouse models.

### 5.7. Inflammation and Hypoglycemia-Induced Dementia

Hypoglycemia induces a plethora of inflammatory markers in the body and is also associated with cognitive impairment. The predominant mechanism is via the production of ROS, leading to two important outcomes: altered immune function and inflammation [114,115]. There is limited information regarding how hypoglycemia causes the generation of ROS; however, studies have noted the dysfunction of the mitochondrial ETC in the brain as the most likely mechanism [116]. 

A recent study conducted in mice demonstrated a causal mechanism between blood–brain barrier (BBB) dysfunction and hypoglycemia, crucial for understanding the resultant pathophysiology, as a previous study reported that AD patients had greater BBB leakage versus a healthy population [117]. This study attributed BBB dysfunction to pericyte damage, due to increased levels of MMP9, an inflammatory pericyte damage marker that, in turn, decreases intracellular tight junction proteins in the BBB. This allows neurotoxic vascular molecules to leak through the BBB and enhance neuronal damage [72].

**Figure 3:**
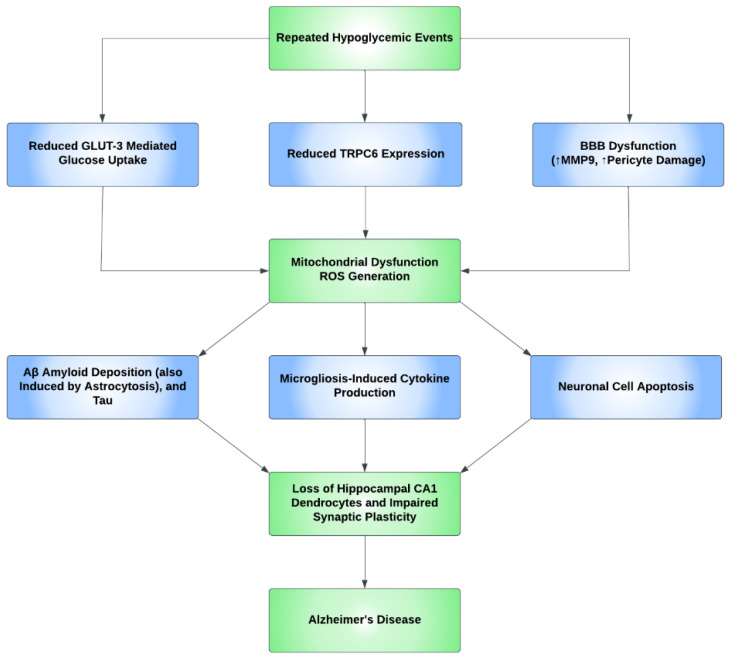
A proposed mechanism for the development of Alzheimer’s disease through repeated hypoglycemic events. Repeated hypoglycemic events were found to reduce transient receptor potential canonical channel 6 (TRPC6) expression and reduce GLUT3-mediated glucose uptake. Hypoglycemia also leads to blood–brain barrier (BBB) dysfunction through pericyte damage and an increase in the MMP9 inflammatory marker. Ultimately, the aforementioned events lead to mitochondrial dysfunction and, subsequently, reactive oxygen species (ROS) generation. ROS then increases beta-amyloid (Aβ) and tau deposition and causes neuronal apoptosis. ROS also causes microglia-induced cytokine production. These events cause the loss of the dendrocytes in the CA1 subregion of the hippocampus and impair synaptic plasticity, which ultimately increases the risk of Alzheimer’s disease (AD).

Oxidative stress causes apoptosis in a number of cell types in the brain. Studies have shown neural cell positivity to a variety of apoptosis and inflammatory markers consequent upon hypoglycemic episodes. It was also demonstrated that the glycemic levels of 30–35 mg/dL increased the number of cells positive for terminal deoxynucleotidyl transferase dUTP nick end labeling (TUNEL), a marker of apoptosis, in the arcuate nucleus of the hypothalamus, and additionally, Fluoro-Jade B (FJB), a marker of degenerating neurons, has also been found in the cerebral cortex, the striatum, and the hippocampus [118]. Additional studies determined that recurrent hypoglycemia causes oxidative injury in hippocampal CA1 dendrocytes, with further microglial activation induced by severe hypoglycemia, contributing to neuronal injury [119]. Furthermore, a study concluded that hypoglycemia accelerates the progress of AD and, through the process of inflammation, induces microgliosis and astrocytosis. Predominant inflammatory markers include IL-1β, IL-6, TNF-α, and IFN-γ [120]. Cumulatively, the aforementioned studies show that a neuroinflammatory process occurs, predominantly in the BBB and hippocampus, resulting in increased BBB permeability, impaired synaptic plasticity, microgliosis and astrocytosis, and CA1 dendritic injury, with the unexpected absence/rarity of neuronal cell death in the hippocampus. Figure 3 shows the proposed mechanism for how AD develops through neuroinflammatory processes secondary to hypoglycemia.

### 5.8. Molecular Factors and Transport Channels

Hypoglycemia can alter the expression of many proteins and molecules in the neurovascular unit, an important one being transient receptor potential canonical channel 6 (TRPC6). A study conducted in mice demonstrated that this protein is sensitive to low glucose concentrations and is hence repressed when exposed to recurrent moderate hypoglycemia, as opposed to higher glucose concentrations, which cause an increase in mRNA and protein expression levels of TRPC6. This decline in TRPC6 expression has been directly associated with significantly reduced fluorescence intensity in neurons and dendrites, as well as positive TUNEL staining, indicative of apoptosis, in the CA1 and CA3 areas of the hippocampus. This demonstrates that TRPC6 inactivation is an aggravating feature for hypoglycemia-induced cognitive impairment, and a protective feature in cases of its activation, making TRPC6 a potential therapeutic target [120,121].

Glucose uptake in neurons is almost solely conducted through GLUT3 channels. Recurrent moderate hypoglycemia is not thought to significantly affect GLUT3 expression, but it does reduce GLUT3-mediated glucose uptake, thus impacting cellular respiration and enhancing mitochondrial dysfunction, which, by extension, inhibits TRPC6 expression [120].

## 6. Therapeutic Options or Treatment Opportunities of Hypoglycemia-Induced Dementia

### 6.1. Continuous Glucose Monitoring and Early Detection of Hypoglycemia in Elderly Patients

CGM has proven to be beneficial in elderly patients who are more susceptible to asymptomatic and nocturnal hypoglycemia [122]. With the use of CGM, an improvement in glycemic control is apparent in patients with T1D irrespective of sociodemographic or economic factors, or the mode of insulin delivery [123]. Studies have revealed that CGM decreased the time spent in hypoglycemia and is associated with a decrease in HbA1c in patients with T1D [32,124]. In comparison to T1D, many patients with T2D struggle to achieve glycemic control. This could be because T2D patients utilize diabetes technologies less frequently than T1D patients [123]. The majority of reports focused on the role of CGM in T2D management and selected trials that employed intensive insulin therapy in T2D, thereby excluding a large number of T2D patients on less intensive regimens. Despite this fact, the available evidence is positive, showing long-term improvement in patient-oriented outcomes in T2D patients using CGM [123]. All T2D patients are advised to self-monitor their glucose levels, as results can help a patient schedule and organize various aspects of their lifestyle to accommodate for significant changes in glucose levels. However, the self-monitoring of glucose levels should not be so intensively performed as to warrant the use of CGM, as there are no patient-oriented advantages to justify CGM’s high cost and added difficulties for both patients and doctors, unless further research is carried out to support its use in T2D patients, particularly when insulin injections are not indicated for the patient [31].

### 6.2. The Importance of Documenting the Events of Hypoglycemia: The Use of Predictive Technologies

In a narrative review written by Heller et al., it was reported that only 26% of the population with T1D and 33% with T2D received medical care for hypoglycemic episodes, with only 24% (T1D) and 31% (T2D) consulting with their own physician. In individuals experiencing frequent hypoglycemic events, there was a 15% underestimation in the rate of recall [125,126]. Examples of technology used to monitor blood glucose levels are self-monitoring blood glucose (SMBG) and interstitial glucose sampling using CGM [127]. Electronic logbooks have also allowed for more robust data collection of hypoglycemic events. These systems, however, do not usually provide details that enable an understanding of the cause, severity of symptoms, or treatments employed in response to a hypoglycemic episode [125]. The main established method of hypoglycemia prediction is using classical time-series forecasting methods on blood glucose data [126]. Examples include the autoregressive integrated moving average (ARIMA) and state–space models. Recently, new machine learning methods have been investigated to predict future glucose levels and have incorporated data such as insulin levels, meal information, physical activity, and heart rate. Despite how these predictive models have helped in reducing hypoglycemia risks, a major hindrance comes from false alerts, and new models are in development to reduce the false alert rate [128].

### 6.3. Should Antidiabetic Drugs Be Continued in Diabetic Patients Who Develop Severe Hypoglycemia-Induced Dementia?

Antidiabetic drugs aim to maintain or reduce blood glucose levels to close-to-normal values for the majority of the time. The mechanism of action of glucose-lowering drugs varies according to drug class, with some able to counteract the insulin resistance and impaired glucose metabolism that can contribute towards dementia and AD [129]. Medications such as metformin, short-acting sulfonylureas, dipeptidyl peptidase 4 (DPP-4) inhibitors, and sodium–glucose cotransporter 2 (SGLT2) inhibitors have a low risk of inducing hypoglycemia [40]. By contrast, long-acting sulfonylureas and insulin have an increased risk of inducing hypoglycemia [40]. There are also drugs such as thiazolidinediones (pioglitazone) that decrease the risk of dementia as well as hypoglycemia [130]. 

In a systematic review conducted by McMillan et al., it was found that, overall, antidiabetic agents were not associated with dementia incidence. However, the results of subanalysis between different drug classes revealed that insulin therapy was linked to dementia risk, whereas thiazolidinediones were potentially protective. Moreover, it was found that a twofold increase in the risk of dementia was associated with severe hypoglycemic episodes [60]. A study by Weinstein et al., which aimed at determining whether antidiabetic drugs are associated with cognitive dysfunction and dementia, revealed that insulin therapy was associated with cognitive dysfunction due to the greater risk of hypoglycemia. By contrast, metformin and sulfonylureas were not associated with dementia risk, nor with other measures of cognitive aging [129]. 

Generally, elderly patients who are at high risk of hypoglycemic events should be prescribed medication that has minimal risk of inducing hypoglycemia. Moreover, polypharmacy should be simplified to reduce adverse effects and achieve appropriate glycemic goals for each patient [40].

Machine learning algorithms predict outputs given input values. They are generally categorized as regression, prediction, classification, detection, and clustering [131]. The most commonly chosen algorithms for hypoglycemia detection and prediction include artificial neural networks (ANNs) [131], and the second most common choice is kernel-based support vector machine (SVM) [126]. In their research, Mosquera-Lopez et al. used a support vector regression (SVR) model considering patients with T1D by training it to predict, before bedtime, any overnight hypoglycemia. The algorithm was able to predict 94.1% of overnight hypoglycemic events, with a 95% confidence interval. This indicates that, when trained using large datasets and optimized via decision theoretic criterion, an SVR model can predict overnight hypoglycemic events and consequently reduce the risk of nocturnal hypoglycemia [132]. Other algorithms include decision trees (DTs), which, through several tree branches, can predict the outcome for a set of input features after testing them [126], and random forests (RFs), which combine several DTs. In addition, quantile regression forests (QRFs), which use a regression approach to predict future CGM values, are being developed [128].

### 6.4. Is Insulin Therapy Still Needed to Protect against Dementia?

Studies have shown that insulin therapy plays a role in preventing AD by promoting a reduction in intracellular amyloid plaque, tau hypophosphorylation, and Aβ-derived diffusible ligand-binding site downregulation. The main insulin therapies are intravenous insulin and intranasal insulin [133]. 

Intravenous insulin increases plasma insulin levels whilst maintaining normal plasma glucose levels, allowing for verbal declarative memory recollection and selective attention enhancement [133]. Studies have shown that, in elderly subjects with AD, insulin infusion improved declarative memory in those who had cognitive impairment in comparison to the healthy control group. Moreover, other studies have shown that insulin infusion improved memory performance in all individuals [134].

Intranasal insulin can be safely given to individuals without diabetes, as it has been tolerated well in clinical tests [135]. A recent systematic review and meta-analysis conducted by Long et al. revealed no significant difference between the use of intranasal insulin and the placebo group in improving cognition in mild cognitive impairment (MCI) or dementia, with the exception of verbal cognition. However, due to the scarcity of clinical trials in this area, no definitive conclusion can be drawn about the effectiveness of intranasal insulin use for dementia protection, although there is interest in its potential efficacy [136].

### 6.5. Effect of Lifestyle Modification in Hypoglycemia-Induced Dementia

Lifestyle modifications can decrease HbA1c levels in patients with T2D. The two main non-pharmacological interventions are dietary changes and physical exercise [137,138].

#### 6.5.1. Dietary Intervention

A diet that is high in simple carbohydrates and saturated fats increases the risk of insulin resistance, T2D, and related cognitive impairment. This occurs due to amyloid precursor protein processing modification through the elevation of Aβ-related cerebrovascular disturbance, a reduction in brain insulin signaling, and the reduced expression of the IDE. 

The Mediterranean diet can decrease HbA1c levels, thus achieving better glycemic control with lower fasting blood glucose levels and therefore decreasing insulin resistance. This, in turn, decreases the risk of age-related cognitive decline, diabetes, and cardiovascular diseases [139]. Several systematic reviews and meta-analyses have shown the benefits of the Mediterranean diet, including a study by Schwingshackl et al. that demonstrated a 19% decrease in risk of T2D in patients who adhered to the Mediterranean diet and another by Koloverou et al. that showed a 23% decrease [140]. Additionally, the consumption of dietary minerals has shown a protective effect against metabolic diseases including T2D; magnesium modifies Aβ polypeptide processing and stimulates the α-secretase cleavage pathway, hence playing a role in cognitive protection. Moreover, magnesium deficiency can lead to oxidative stress, thereby contributing to AD pathology [137].

#### 6.5.2. Physical Exercise

Physical exercise has been shown to improve learning and memory in humans and decrease the risk of dementia [141]. Different types of physical activity act as an effective treatment for diabetes. First, regular exercise has been found to lower brain Aβ deposition [139], blood pressure, insulin resistance, dyslipidemia, and HbA1c in T2D individuals [141]. Aerobic exercise, at moderate or high intensity, increases insulin sensitivity, cardiac output, blood vessel compliance and responsiveness, pulmonary function, and immunological performance in both T1D and T2D. In addition, it decreases HbA1c [141], consequently decreasing cardiovascular mortality rates in diabetic patients and allowing for better glycemic control. However, patients should avoid exercise during episodes of hypoglycemia and immediately afterward (for 24 h), as moderately intensive exercise can induce hypoglycemia [142].

### 6.6. Hypoglycemia Risk Minimization in Patients with Type 2 Diabetes

Both pharmacological and non-pharmacological strategies have been introduced to try to break the cycle of hypoglycemia-induced dementia. From a non-pharmacological perspective, lifestyle modifications as well as the implementation of structured care tailored to the patient’s individual needs can help maintain therapeutic glycemic goals [143].

Pharmacological risk reduction has been facilitated by the use of newly emerging antidiabetic therapies such as incretin enhancement therapies (incretin analogs and dipeptidyl–peptidase 4 inhibitors) instead of sulfonylureas and insulin secretagogues [144]. Patients utilizing lifestyle adjustment strategies and/or metformin and peroxisome proliferator-activated receptor (PPAR) agonists have shown a relatively negligible risk for hypoglycemia. The UKPDS 73 study showed a 0.3% and 0.1% hypoglycemic risk rate for metformin and lifestyle modification in patients receiving monotherapy or dietary modifications, respectively, for 6 years after diagnosis [145]. Additionally, the ADOPT study showed a lower risk of hypoglycemia with rosiglitazone and metformin than with glyburide over 5 years of treatment [146]. The UKPDS 33 study examined the risk of hypoglycemia in patients initially treated with insulin therapy, reporting hypoglycemia with “any” initial insulin therapy had rates of 33% and 43% at years 1 and 10, respectively [147]. Furthermore, data acquired from the DARTS-MEMO database shows that at least one episode of severe hypoglycemia in insulin-treated patients arose in about 7.3% of T2D and 7.1% of T1D patients [148]. Sulfonylureas similarly showed a higher risk for hypoglycemic episodes, with a United Kingdom hypoglycemia study commissioned by the Department for Transport showing hypoglycemic self-reported rates of 39% (mild) and 7% (severe) in patients with T2D on sulfonylureas [149]. However, studies have shown that treatment with new third-generation sulfonylureas (glimepiride, glipizide, and gliclazide) and metiglinides lowers the risk versus glibenclamide and chlorpropamide. Other contributory factors, such as differing duration of action, are thought to contribute to this difference [150]. 

A Canadian population-based study demonstrated how the introduction of new antidiabetic drugs has changed trends of antidiabetic medication prescriptions between 2002 and 2013 in terms of lowering the risk of hypoglycemia [151]. Trends show increased prescriptions for metformin and new medications, and decreased use of glyburide. Physicians preferred a combination of different medications, especially in newly diagnosed patients. These changes were consistent with the clinical guidelines that encouraged the use of medications with lower hypoglycemia risk and, over the study period, the overall percentage of hypoglycemia declined [152]. Newer agents seem to have a favorable impact on cerebral protection, through direct and indirect mechanisms, as has been reported in multiple studies [152,153,154,155].

### 6.7. Management of Hypoglycemia in Older Patients with Type 2 Diabetes

Due to comorbidities increasing with age, a personalized approach for glycemic targets is needed to achieve adequate glucose control and avoid T2D complications [40]. The American College of Physicians recommends less intensive medication regimens when HbA1c levels are 6.5% or below and more conservative goals when HbA1c values are between 7% and 8%. The American Association of Clinical Endocrinologists recommends HbA1c levels less than 6.5%, as long as this level can be achieved safely, with a more lenient target (>6.5%) for diabetic patients with comorbid illnesses [40].

Before pharmacological intervention, lifestyle modifications should be implemented to reduce hypoglycemic events in elderly patients. These include a balanced diet and undertaking moderate regular physical exercise, with regular checks of blood pressure and lipid levels [156]. Establishing patient adherence to prescribed medication is crucial [156]. Since elderly patients are more likely to be taking more than one medication, drug costs may create a financial burden. Therefore, treatment goals should be developed and achieved according to the medical, social, and economic status of elderly patients [157]. Pharmacological intervention to reduce the frequency of hypoglycemic episodes should be applied to the elderly. The first line of treatment for elderly patients with an estimated glomerular filtration rate of ≥30 mL/min/1.73 is metformin, as it has a low risk of causing hypoglycemia and has demonstrated cardiovascular safety.

Future interventions look promising. A study by Suh et al. showed that poly(ADP-ribose)-polymerase-1 (PARP-1) inhibitors immediately blocked the production of poly(ADP-ribose) and decreased neuronal death by greater than 80% in the majority of brain regions examined after hypoglycemia [158]. PARP-1 has been demonstrated to be involved in neurodegenerative conditions and aging, via its ability to induce “parthanatos”, a unique cell death pathway, and subsequently impairs autophagy and leads to an accumulation of neurotoxic peptides, and PARP-1 has also been implicated to play a role in DNA excision repair, neuroinflammation, and catalyzing DNA NF-KB binding in microglia [159,160]. Hence, PARP-1 inhibitors can delay AD progression through the mitigation of microglia activation, which was shown in mouse models [161].

Furthermore, as TRPC6 dysfunction has been closely linked with cognitive impairment in T2D patients with recurrent episodes of mild hypoglycemia, a promising study in mice revealed that targeting TRPC6 was a therapeutic option for treating hypoglycemia-induced cognitive dysfunction [121]. It was found that upon the activation of TRPC6 with hyperforin, mitochondrial segregation was halted in the hippocampi of diabetic mice, via an increase in TRPC6-mediated calcium influx and the subsequent activation of adenosine 5‘-monophosphate-activated protein kinase (AMPK) [121].

### 6.8. Role of Personalized Medicine in Managing Hypoglycemia and Associated Complications

Personalized medicine is an emerging concept in disease treatment that involves determining a patient’s treatment by using specific information about a particular patient’s social, economic, and medical status. In the case of diabetes, personalized medicine takes into account one’s genetic makeup to tailor prevention, detection, treatment, and monitoring strategies [162], thereby increasing treatment effectiveness whilst reducing treatment failures and adverse effects [163].

One example of personalized medicine is in patients with monogenic diabetes. Monogenic diabetes is caused by a defect in a single gene and causes ~1% of all diabetes. The most well-known and well-studied form is maturity-onset diabetes of the young (MODY). Patients with MODY1 and MODY3 have been found to be extremely sensitive to sulfonylureas; therefore, these patients should be treated with sulfonylureas as their first-line treatment and require approximately one-tenth of the usual sulfonylureas dose to maintain optimal HbA1c levels [163].

Personalized medicine requires that each patient’s genotype be analyzed, a costly endeavor, though this is likely to decrease in the future. Other barriers include training an adequate number of genetics specialists in order to optimally apply the results of genetic testing, obtaining adequate insurance coverage, and guaranteeing the privacy of genetic records [162].

## 7. Conclusions

Taken as a whole, a comprehensive understanding of the role of hypoglycemia in the progression of dementia is crucial to determining effective preventive measures and tackling the health burden of the disease. Hypoglycemia plays a definitive role in cognitive impairment and the progression of dementia in both type 1 and type 2 diabetic patients. The pathological relationship between the two is complex, with the available literature suggesting common pathological abnormalities such as insulin resistance, increased oxidative stress, and impaired glucose metabolism. Additionally, the deposition of amyloid proteins, and diabetes risk-induced micro- and macrovascular changes are also key pathological abnormalities when considering the relationship between the two.

Hypoglycemic cognitive decline is a result of a variety of complex mechanistic pathways explored in the literature, such as ROS generation in the brain, blood–brain barrier damage, and the effects of chronic hyperinsulinemia. Furthermore, insulin receptor resistance leading to dysfunction, and cerebral small vessel disease are also important when discussing the complex pathways contributing to hypoglycemic cognitive decline. 

There are many risk factors for hypoglycemia-induced dementia in diabetics, with age and genetics being the most prominent. The elderly population is specifically at risk, and it is important to identify risk factors of hypoglycemia, such as lifestyle and polypharmacy, to allow for early intervention and a reduction in dementia risk. Newer generation antidiabetic therapies are reported to have a lower incidence of drug-induced hypoglycemia than standard insulin replacement therapy and are therefore preferentially used in current medical practice. Predictive technologies that use current glucose monitoring systems to predict hypoglycemia and the evolution of personalized medicine have raised glucose monitoring and patient treatment to a new level of precision and have also facilitated the reduction in hypoglycemia-induced dementia. Recent reports also suggest that the future use of drug targets such as TRPC6 could be promising in hypoglycemic episode prevention.

## Figures and Tables

**Table 1:** Systematic reviews noting risk ratios between hypoglycemia and dementia.

Title of Study	Number of Hypoglycemic Events	Pooled Risk Ratio (95% CI) for Association between Hypoglycemia and Dementia	Reference
Association of Hypoglycemia with Subsequent Dementia in Older Patients with Type 2 Diabetes Mellitus	≥1	1.27 (1.06, 1.51)	[58]
Association between Hypoglycemia and Risk of Dementia in Patients with Type 2 Diabetes Mellitus: A Systematic Review and Meta-Analysis	1	1.29 (1.15, 1.44)	[59]
	2	1.68 (1.38, 2.04)	
	3	1.99 (1.48, 2.68)	
Impact of Pharmacological Treatment of Diabetes Mellitus on Dementia Risk: Systematic Review and Meta-Analysis	≥1	1.77 (1.35, 2.33)	[60]

## Data Availability

No new data were generated in the writing of this review article.

## References

[1] American Diabetes Association (2018). 6. Glycemic Targets: Standards of Medical Care in Diabetes—2018. Diabetes Care.

[2] Huang L., Zhu M., Ji J. (2022). Association between Hypoglycemia and Dementia in Patients with Diabetes: A Systematic Review and Meta-Analysis of 1.4 Million Patients. Diabetol. Metab. Syndr..

[3] Amiel S.A. (2021). The Consequences of Hypoglycaemia. Diabetologia.

[4] Yanai H., Adachi H., Katsuyama H., Moriyama S., Hamasaki H., Sako A. (2015). Causative Anti-Diabetic Drugs and the Underlying Clinical Factors for Hypoglycemia in Patients with Diabetes. World J. Diabetes.

[5] Sprague J.E., Arbeláez A.M. (2011). Glucose Counterregulatory Responses to Hypoglycemia. Pediatr. Endocrinol. Rev..

[6] Cooperberg B.A., Cryer P.E. (2010). Insulin Reciprocally Regulates Glucagon Secretion in Humans. Diabetes.

[7] Nakhleh A., Shehadeh N. (2021). Hypoglycemia in Diabetes: An Update on Pathophysiology, Treatment, and Prevention. World J. Diabetes.

[8] Gangji A.S., Cukierman T., Gerstein H.C., Goldsmith C.H., Clase C.M. (2007). A Systematic Review and Meta-Analysis of Hypoglycemia and Cardiovascular Events: A Comparison of Glyburide with Other Secretagogues and with Insulin. Diabetes Care.

[9] Meneilly G.S., Cheung E., Tuokko H. (1994). Counterregulatory Hormone Responses to Hypoglycemia in the Elderly Patient with Diabetes. Diabetes.

[10] Bremer J.P., Jauch-Chara K., Hallschmid M., Schmid S., Schultes B. (2009). Hypoglycemia Unawareness in Older Compared with Middle-Aged Patients with Type 2 Diabetes. Diabetes Care.

[11] Lopez O.L., Kuller L.H. (2019). Epidemiology of Aging and Associated Cognitive Disorders: Prevalence and Incidence of Alzheimer’s Disease and Other Dementias. Handb. Clin. Neurol..

[12] Kane J.P.M., Surendranathan A., Bentley A., Barker S.A.H., Taylor J.-P., Thomas A.J., Allan L.M., McNally R.J., James P.W., McKeith I.G. (2018). Clinical Prevalence of Lewy Body Dementia. Alzheimer’s Res. Ther..

[13] Gudala K., Bansal D., Schifano F., Bhansali A. (2013). Diabetes Mellitus and Risk of Dementia: A Meta-Analysis of Prospective Observational Studies. J. Diabetes Investig..

[14] Epidemiology of Severe Hypoglycemia in the Diabetes Control and Complications Trial (1991). The DCCT Research Group. Am. J. Med..

[15] Cryer P.E. (2005). Mechanisms of Hypoglycemia-Associated Autonomic Failure and Its Component Syndromes in Diabetes. Diabetes.

[16] Donnelly L.A., Morris A.D., Frier B.M., Ellis J.D., Donnan P.T., Durrant R., Band M.M., Reekie G., Leese G.P. (2005). DARTS/MEMO Collaboration Frequency and Predictors of Hypoglycaemia in Type 1 and Insulin-Treated Type 2 Diabetes: A Population-Based Study. Diabet. Med..

[17] Miech R.A., Breitner J.C.S., Zandi P.P., Khachaturian A.S., Anthony J.C., Mayer L. (2002). Incidence of AD May Decline in the Early 90s for Men, Later for Women: The Cache County Study. Neurology.

[18] McAulay V., Deary I.J., Frier B.M. (2001). Symptoms of Hypoglycaemia in People with Diabetes. Diabet. Med..

[19] Kawamura T., Umemura T., Hotta N. (2012). Cognitive Impairment in Diabetic Patients: Can Diabetic Control Prevent Cognitive Decline?. J. Diabetes Investig..

[20] Cukierman-Yaffe T., Gerstein H.C., Williamson J.D., Lazar R.M., Lovato L., Miller M.E., Coker L.H., Murray A., Sullivan M.D., Marcovina S.M. (2009). Relationship between Baseline Glycemic Control and Cognitive Function in Individuals with Type 2 Diabetes and Other Cardiovascular Risk Factors: The Action to Control Cardiovascular Risk in Diabetes-Memory in Diabetes (ACCORD-MIND) Trial. Diabetes Care.

[21] Coucha M., Abdelsaid M., Ward R., Abdul Y., Ergul A. (2018). Impact of Metabolic Diseases on Cerebral Circulation: Structural and Functional Consequences. Compr. Physiol..

[22] Salim S. (2017). Oxidative Stress and the Central Nervous System. J. Pharmacol. Exp. Ther..

[23] Singh V.P., Bali A., Singh N., Jaggi A.S. (2014). Advanced Glycation End Products and Diabetic Complications. Korean J. Physiol. Pharmacol..

[24] Nowotny K., Jung T., Höhn A., Weber D., Grune T. (2015). Advanced Glycation End Products and Oxidative Stress in Type 2 Diabetes Mellitus. Biomolecules.

[25] Ravona-Springer R., Schnaider-Beeri M. (2011). The Association of Diabetes and Dementia and Possible Implications for Nondiabetic Populations. Expert Rev. Neurother..

[26] Dos Santos Matioli M.N.P., Suemoto C.K., Rodriguez R.D., Farias D.S., da Silva M.M., Leite R.E.P., Ferretti-Rebustini R.E.L., Farfel J.M., Pasqualucci C.A., Jacob Filho W. (2017). Diabetes Is Not Associated with Alzheimer’s Disease Neuropathology. J. Alzheimer’s Dis..

[27] Feinkohl I., Price J.F., Strachan M.W.J., Frier B.M. (2015). The Impact of Diabetes on Cognitive Decline: Potential Vascular, Metabolic, and Psychosocial Risk Factors. Alzheimer’s Res. Ther..

[28] Kwa V.I.H., van der Sande J.J., Stam J., Tijmes N., Vrooland J.L. (2002). Amsterdam Vascular Medicine Group Retinal Arterial Changes Correlate with Cerebral Small-Vessel Disease. Neurology.

[29] Matsuda M., Shimomura I. (2013). Increased Oxidative Stress in Obesity: Implications for Metabolic Syndrome, Diabetes, Hypertension, Dyslipidemia, Atherosclerosis, and Cancer. Obes. Res. Clin. Pract..

[30] Czupryniak L., Dzida G., Fichna P., Jarosz-Chobot P., Gumprecht J., Klupa T., Mysliwiec M., Szadkowska A., Bomba-Opon D., Czajkowski K. (2022). Ambulatory Glucose Profile (AGP) Report in Daily Care of Patients with Diabetes: Practical Tips and Recommendations. Diabetes Ther..

[31] Robertson S.L., Shaughnessy A.F., Slawson D.C. (2020). Continuous Glucose Monitoring in Type 2 Diabetes Is Not Ready for Widespread Adoption. Am. Fam. Physician.

[32] Pratley R.E., Kanapka L.G., Rickels M.R., Ahmann A., Aleppo G., Beck R., Bhargava A., Bode B.W., Carlson A., Chaytor N.S. (2020). Effect of Continuous Glucose Monitoring on Hypoglycemia in Older Adults With Type 1 Diabetes: A Randomized Clinical Trial. JAMA.

[33] Kröger J., Reichel A., Siegmund T., Ziegler R. (2020). Clinical Recommendations for the Use of the Ambulatory Glucose Profile in Diabetes Care. J. Diabetes Sci. Technol..

[34] Vigersky R.A., McMahon C. (2019). The Relationship of Hemoglobin A1C to Time-in-Range in Patients with Diabetes. Diabetes Technol. Ther..

[35] Beck R.W., Bergenstal R.M., Riddlesworth T.D., Kollman C., Li Z., Brown A.S., Close K.L. (2019). Validation of Time in Range as an Outcome Measure for Diabetes Clinical Trials. Diabetes Care.

[36] Lu J., Wang C., Shen Y., Chen L., Zhang L., Cai J., Lu W., Zhu W., Hu G., Xia T. (2021). Time in Range in Relation to All-Cause and Cardiovascular Mortality in Patients With Type 2 Diabetes: A Prospective Cohort Study. Diabetes Care.

[37] (2012). American Geriatrics Society Expert Panel on the Care of Older Adults with Multimorbidity Patient-Centered Care for Older Adults with Multiple Chronic Conditions: A Stepwise Approach from the American Geriatrics Society: American Geriatrics Society Expert Panel on the Care of Older Adults with Multimorbidity. J. Am. Geriatr. Soc..

[38] Kalyani R.R., Egan J.M. (2013). Diabetes and Altered Glucose Metabolism with Aging. Endocrinol. Metab. Clin. N. Am..

[39] American Diabetes Association (2019). 2. Classification and Diagnosis of Diabetes: Standards of Medical Care in Diabetes—2019. Diabetes Care.

[40] Longo M., Bellastella G., Maiorino M.I., Meier J.J., Esposito K., Giugliano D. (2019). Diabetes and Aging: From Treatment Goals to Pharmacologic Therapy. Front. Endocrinol..

[41] Moheet A., Mangia S., Seaquist E.R. (2015). Impact of Diabetes on Cognitive Function and Brain Structure. Ann. N. Y. Acad. Sci..

[42] Correia S.C., Santos R.X., Carvalho C., Cardoso S., Candeias E., Santos M.S., Oliveira C.R., Moreira P.I. (2012). Insulin Signaling, Glucose Metabolism and Mitochondria: Major Players in Alzheimer’s Disease and Diabetes Interrelation. Brain Res..

[43] De la Monte S.M., Wands J.R. (2008). Alzheimer’s Disease Is Type 3 Diabetes-Evidence Reviewed. J. Diabetes Sci. Technol..

[44] Rojas M., Chávez-Castillo M., Bautista J., Ortega Á., Nava M., Salazar J., Díaz-Camargo E., Medina O., Rojas-Quintero J., Bermúdez V. (2021). Alzheimer’s Disease and Type 2 Diabetes Mellitus: Pathophysiologic and Pharmacotherapeutics Links. World J. Diabetes.

[45] Convit A. (2005). Links between Cognitive Impairment in Insulin Resistance: An Explanatory Model. Neurobiol. Aging.

[46] Crane P.K., Walker R., Hubbard R.A., Li G., Nathan D.M., Zheng H., Haneuse S., Craft S., Montine T.J., Kahn S.E. (2013). Glucose Levels and Risk of Dementia. N. Engl. J. Med..

[47] Benveniste H., Liu X., Koundal S., Sanggaard S., Lee H., Wardlaw J. (2019). The Glymphatic System and Waste Clearance with Brain Aging: A Review. Gerontology.

[48] Zhang L., Chopp M., Jiang Q., Zhang Z. (2019). Role of the Glymphatic System in Ageing and Diabetes Mellitus Impaired Cognitive Function. Stroke Vasc. Neurol..

[49] Jiang Q., Zhang L., Ding G., Davoodi-Bojd E., Li Q., Li L., Sadry N., Nedergaard M., Chopp M., Zhang Z. (2017). Impairment of the Glymphatic System after Diabetes. J. Cereb. Blood Flow Metab..

[50] Reddy O.C., van der Werf Y.D. (2020). The Sleeping Brain: Harnessing the Power of the Glymphatic System through Lifestyle Choices. Brain Sci..

[51] Pugazhenthi S., Qin L., Reddy P.H. (2017). Common Neurodegenerative Pathways in Obesity, Diabetes, and Alzheimer’s Disease. Biochim. Biophys. Acta Mol. Basis Dis..

[52] Chen L., Chen R., Wang H., Liang F. (2015). Mechanisms Linking Inflammation to Insulin Resistance. Int. J. Endocrinol..

[53] Xu W.L., Atti A.R., Gatz M., Pedersen N.L., Johansson B., Fratiglioni L. (2011). Midlife Overweight and Obesity Increase Late-Life Dementia Risk: A Population-Based Twin Study. Neurology.

[54] Alwafi H., Alsharif A.A., Wei L., Langan D., Naser A.Y., Mongkhon P., Bell J.S., Ilomaki J., Al Metwazi M.S., Man K.K.C. (2020). Incidence and Prevalence of Hypoglycaemia in Type 1 and Type 2 Diabetes Individuals: A Systematic Review and Meta-Analysis. Diabetes Res. Clin. Pract..

[55] Shuba N. (2012). Karan Assessment of the Cognitive Status in Diabetes Mellitus. J. Clin. Diagn. Res..

[56] Naguib R., Soliman E.S., Neimatallah F.M., AlKhudhairy N.S., ALGhamdi A.M., Almosa R.S., Aldashash K.A., Alkhalifah B.Y., Elmorshedy H. (2020). Cognitive Impairment among Patients with Diabetes in Saudi Arabia: A Cross-Sectional Study. Middle East Curr. Psychiatry.

[57] Alsharif A.A., Wei L., Ma T., Man K.K.C., Lau W.C.Y., Brauer R., Almetwazi M., Howard R., Wong I.C.K. (2020). Prevalence and Incidence of Dementia in People with Diabetes Mellitus. J. Alzheimer’s Dis..

[58] Mehta H.B., Mehta V., Goodwin J.S. (2019). Association of Hypoglycemia with Subsequent Dementia in Older Patients with Type 2 Diabetes Mellitus. J. Gerontol. A Biol. Sci. Med. Sci..

[59] Gómez-Guijarro M.D., Álvarez-Bueno C., Saz-Lara A., Sequí-Domínguez I., Lucerón-Lucas-Torres M., Cavero-Redondo I. (2023). Association between severe hypoglycaemia and risk of dementia in patients with type 2 diabetes mellitus: A systematic review and meta-analysis. Diabetes Metab. Res. Rev..

[60] McMillan J.M., Mele B.S., Hogan D.B., Leung A.A. (2018). Impact of Pharmacological Treatment of Diabetes Mellitus on Dementia Risk: Systematic Review and Meta-Analysis. BMJ Open Diabetes Res. Care.

[61] Kim Y.G., Park D.G., Moon S.Y., Jeon J.Y., Kim H.J., Kim D.J., Lee K.W., Han S.J. (2020). Hypoglycemia and Dementia Risk in Older Patients with Type 2 Diabetes Mellitus: A Propensity-Score Matched Analysis of a Population-Based Cohort Study. Diabetes Metab. J..

[62] Whitmer R.A., Karter A.J., Yaffe K., Quesenberry C.P., Selby J.V. (2009). Hypoglycemic Episodes and Risk of Dementia in Older Patients with Type 2 Diabetes Mellitus. JAMA.

[63] Zheng B., Su B., Price G., Tzoulaki I., Ahmadi-Abhari S., Middleton L. (2021). Glycemic Control, Diabetic Complications, and Risk of Dementia in Patients With Diabetes: Results From a Large U.K. Cohort Study. Diabetes Care.

[64] Tang X., Cardoso M.A., Yang J., Zhou J.-B., Simó R. (2021). Impact of Intensive Glucose Control on Brain Health: Meta-Analysis of Cumulative Data from 16,584 Patients with Type 2 Diabetes Mellitus. Diabetes Ther..

[65] Inkster B., Zammitt N.N., Frier B.M. (2012). Drug-Induced Hypoglycaemia in Type 2 Diabetes. Expert Opin. Drug Saf..

[66] Xie L., Zhou S., Pinsky B.W., Buysman E.K., Baser O. (2014). Impact of Initiating Insulin Glargine Disposable Pen versus Vial/syringe on Real-World Glycemic Outcomes and Persistence among Patients with Type 2 Diabetes Mellitus in a Large Managed Care Plan: A Claims Database Analysis. Diabetes Technol. Ther..

[67] Asche C.V., Luo W., Aagren M. (2013). Differences in Rates of Hypoglycemia and Health Care Costs in Patients Treated with Insulin Aspart in Pens versus Vials. Curr. Med. Res. Opin..

[68] So W.Y., Chan J.C.N., Yeung V.T.F., Chow C.C., Ko G.T.C., Li J.K.Y., Cockram C.S. (2002). Sulphonylurea-Induced Hypoglycaemia in Institutionalized Elderly in Hong Kong. Diabet. Med..

[69] Kong A.P.S., Yang X., Luk A., Cheung K.K.T., Ma R.C.W., So W.Y., Ho C.S., Chan M.H.M., Ozaki R., Chow C.C. (2014). Hypoglycaemia, Chronic Kidney Disease and Death in Type 2 Diabetes: The Hong Kong Diabetes Registry. BMC Endocr. Disord..

[70] Kong A.P.S., Chan J.C.N. (2015). Hypoglycemia and Comorbidities in Type 2 Diabetes. Curr. Diab. Rep..

[71] Saik O.V., Klimontov V.V. (2021). Hypoglycemia, Vascular Disease and Cognitive Dysfunction in Diabetes: Insights from Text Mining-Based Reconstruction and Bioinformatics Analysis of the Gene Networks. Int. J. Mol. Sci..

[72] Lin L., Wu Y., Chen Z., Huang L., Wang L., Liu L. (2021). Severe Hypoglycemia Contributing to Cognitive Dysfunction in Diabetic Mice Is Associated With Pericyte and Blood-Brain Barrier Dysfunction. Front. Aging Neurosci..

[73] Yaffe K., Falvey C.M., Hamilton N., Harris T.B., Simonsick E.M., Strotmeyer E.S., Shorr R.I., Metti A., Schwartz A.V. (2013). Health ABC Study Association between Hypoglycemia and Dementia in a Biracial Cohort of Older Adults with Diabetes Mellitus. JAMA Intern. Med..

[74] Mattishent K., Loke Y.K. (2021). Meta-Analysis: Association Between Hypoglycemia and Serious Adverse Events in Older Patients Treated With Glucose-Lowering Agents. Front. Endocrinol..

[75] Mattishent K., Loke Y.K. (2016). Bi-Directional Interaction between Hypoglycaemia and Cognitive Impairment in Elderly Patients Treated with Glucose-Lowering Agents: A Systematic Review and Meta-Analysis. Diabetes Obes. Metab..

[76] Sun X.J., Liu F. (2009). Phosphorylation of IRS Proteins Yin-Yang Regulation of Insulin Signaling. Vitam. Horm..

[77] Duarte A.I., Santos M.S., Seiça R., de Oliveira C.R. (2003). Insulin Affects Synaptosomal GABA and Glutamate Transport under Oxidative Stress Conditions. Brain Res..

[78] Duarte A.I., Proença T., Oliveira C.R., Santos M.S., Rego A.C. (2006). Insulin Restores Metabolic Function in Cultured Cortical Neurons Subjected to Oxidative Stress. Diabetes.

[79] Ryu B.R., Ko H.W., Jou I., Noh J.S., Gwag B.J. (1999). Phosphatidylinositol 3-Kinase-Mediated Regulation of Neuronal Apoptosis and Necrosis by Insulin and IGF-I. J. Neurobiol..

[80] Garg R., Chaudhuri A., Munschauer F., Dandona P. (2006). Hyperglycemia, Insulin, and Acute Ischemic Stroke: A Mechanistic Justification for a Trial of Insulin Infusion Therapy. Stroke.

[81] Dandona P. (2002). Endothelium, Inflammation, and Diabetes. Curr. Diab. Rep..

[82] Rensink A.A.M., Otte-Höller I., de Boer R., Bosch R.R., ten Donkelaar H.J., de Waal R.M.W., Verbeek M.M., Kremer B. (2004). Insulin Inhibits Amyloid Beta-Induced Cell Death in Cultured Human Brain Pericytes. Neurobiol. Aging.

[83] De la Monte S.M. (2012). Brain Insulin Resistance and Deficiency as Therapeutic Targets in Alzheimer’s Disease. Curr. Alzheimer Res..

[84] Zhao W.Q., Alkon D.L. (2001). Role of Insulin and Insulin Receptor in Learning and Memory. Mol. Cell. Endocrinol..

[85] Craft S., Peskind E., Schwartz M.W., Schellenberg G.D., Raskind M., Porte D. (1998). Cerebrospinal Fluid and Plasma Insulin Levels in Alzheimer’s Disease: Relationship to Severity of Dementia and Apolipoprotein E Genotype. Neurology.

[86] Marks J.L., Porte D., Stahl W.L., Baskin D.G. (1990). Localization of Insulin Receptor mRNA in Rat Brain by in Situ Hybridization. Endocrinology.

[87] Werther G.A., Hogg A., Oldfield B.J., McKinley M.J., Figdor R., Allen A.M., Mendelsohn F.A. (1987). Localization and Characterization of Insulin Receptors in Rat Brain and Pituitary Gland Using in Vitro Autoradiography and Computerized Densitometry. Endocrinology.

[88] Umegaki H., Kawamura T., Umemura T., Kawano N. (2015). Factors Associated with Cognitive Decline in Older Adults with Type 2 Diabetes Mellitus during a 6-Year Observation. Geriatr. Gerontol. Int..

[89] Cole G.M., Frautschy S.A. (2007). The Role of Insulin and Neurotrophic Factor Signaling in Brain Aging and Alzheimer’s Disease. Exp. Gerontol..

[90] Biessels G.J., Kamal A., Ramakers G.M., Urban I.J., Spruijt B.M., Erkelens D.W., Gispen W.H. (1996). Place Learning and Hippocampal Synaptic Plasticity in Streptozotocin-Induced Diabetic Rats. Diabetes.

[91] Sun P., Ortega G., Tan Y., Hua Q., Riederer P.F., Deckert J., Schmitt-Böhrer A.G. (2018). Streptozotocin Impairs Proliferation and Differentiation of Adult Hippocampal Neural Stem Cells in Vitro-Correlation with Alterations in the Expression of Proteins Associated With the Insulin System. Front. Aging Neurosci..

[92] Zhao W., Chen H., Xu H., Moore E., Meiri N., Quon M.J., Alkon D.L. (1999). Brain Insulin Receptors and Spatial Memory. Correlated Changes in Gene Expression, Tyrosine Phosphorylation, and Signaling Molecules in the Hippocampus of Water Maze Trained Rats. J. Biol. Chem..

[93] Kwon H., Pessin J.E. (2013). Adipokines Mediate Inflammation and Insulin Resistance. Front. Endocrinol..

[94] Stranahan A.M., Norman E.D., Lee K., Cutler R.G., Telljohann R.S., Egan J.M., Mattson M.P. (2008). Diet-Induced Insulin Resistance Impairs Hippocampal Synaptic Plasticity and Cognition in Middle-Aged Rats. Hippocampus.

[95] Kelly S.J., Ismail M. (2015). Stress and Type 2 Diabetes: A Review of How Stress Contributes to the Development of Type 2 Diabetes. Annu. Rev. Public Health.

[96] Bi T., Zhan L., Zhou W., Sui H. (2020). Effect of the ZiBuPiYin Recipe on Diabetes-Associated Cognitive Decline in Zucker Diabetic Fatty Rats After Chronic Psychological Stress. Front. Psychiatry.

[97] Medina-Vera D., Navarro J.A., Rivera P., Rosell-Valle C., Gutiérrez-Adán A., Sanjuan C., López-Gambero A.J., Tovar R., Suárez J., Pavón F.J. (2022). D-Pinitol Promotes Tau Dephosphorylation through a Cyclin-Dependent Kinase 5 Regulation Mechanism: A New Potential Approach for Tauopathies?. Br. J. Pharmacol..

[98] Medina-Vera D., Navarro J.A., Tovar R., Rosell-Valle C., Gutiérrez-Adan A., Ledesma J.C., Sanjuan C., Pavón F.J., Baixeras E., Rodríguez de Fonseca F. (2021). Activation of PI3K/Akt Signaling Pathway in Rat Hypothalamus Induced by an Acute Oral Administration of D-Pinitol. Nutrients.

[99] Reddy V.P., Zhu X., Perry G., Smith M.A. (2009). Oxidative Stress in Diabetes and Alzheimer’s Disease. J. Alzheimer’s Dis..

[100] Hollensworth S.B., Shen C., Sim J.E., Spitz D.R., Wilson G.L., LeDoux S.P. (2000). Glial Cell Type-Specific Responses to Menadione-Induced Oxidative Stress. Free Radic. Biol. Med..

[101] Brookes P.S., Yoon Y., Robotham J.L., Anders M.W., Sheu S.-S. (2004). Calcium, ATP, and ROS: A Mitochondrial Love-Hate Triangle. Am. J. Physiol. Cell Physiol..

[102] Cho D.-H., Nakamura T., Fang J., Cieplak P., Godzik A., Gu Z., Lipton S.A. (2009). S-Nitrosylation of Drp1 Mediates Beta-Amyloid-Related Mitochondrial Fission and Neuronal Injury. Science.

[103] Bell S.M., Barnes K., De Marco M., Shaw P.J., Ferraiuolo L., Blackburn D.J., Venneri A., Mortiboys H. (2021). Mitochondrial Dysfunction in Alzheimer’s Disease: A Biomarker of the Future?. Biomedicines.

[104] Zhu X., Lee H.-G., Perry G., Smith M.A. (2007). Alzheimer Disease, the Two-Hit Hypothesis: An Update. Biochim. Biophys. Acta.

[105] Hanson A.J., Banks W.A., Hernandez Saucedo H., Craft S. (2016). Apolipoprotein E Genotype and Sex Influence Glucose Tolerance in Older Adults: A Cross-Sectional Study. Dement. Geriatr. Cogn. Dis. Extra.

[106] Hao J.-S., Zhu C.-J., Yan B.-Y., Yan C.-Y., Ling R. (2018). Stimulation of KLF14/PLK1 Pathway by Thrombin Signaling Potentiates Endothelial Dysfunction in Type 2 Diabetes Mellitus. Biomed. Pharmacother..

[107] Machida T., Takata F., Matsumoto J., Miyamura T., Hirata R., Kimura I., Kataoka Y., Dohgu S., Yamauchi A. (2017). Contribution of Thrombin-Reactive Brain Pericytes to Blood-Brain Barrier Dysfunction in an in Vivo Mouse Model of Obesity-Associated Diabetes and an in Vitro Rat Model. PLoS ONE.

[108] Coughlin S.R. (2005). Protease-Activated Receptors in Hemostasis, Thrombosis and Vascular Biology. J. Thromb. Haemost..

[109] Rahadian A., Fukuda D., Salim H.M., Yagi S., Kusunose K., Yamada H., Soeki T., Shimabukuro M., Sata M. (2020). Thrombin Inhibition by Dabigatran Attenuates Endothelial Dysfunction in Diabetic Mice. Vascul. Pharmacol..

[110] Grammas P., Martinez J., Sanchez A., Yin X., Riley J., Gay D., Desobry K., Tripathy D., Luo J., Evola M. (2014). A New Paradigm for the Treatment of Alzheimer’s Disease: Targeting Vascular Activation. J. Alzheimer’s Dis..

[111] Shlobin N.A., Har-Even M., Itsekson-Hayosh Z.E., Harnof S., Pick C.G. (2021). Role of Thrombin in Central Nervous System Injury and Disease. Biomolecules.

[112] Abu El-Asrar A.M., Alam K., Nawaz M.I., Mohammad G., Van den Eynde K., Siddiquei M.M., Mousa A., De Hertogh G., Opdenakker G. (2016). Upregulation of Thrombin/Matrix Metalloproteinase-1/Protease-Activated Receptor-1 Chain in Proliferative Diabetic Retinopathy. Curr. Eye Res..

[113] Zamolodchikov D., Chen Z.-L., Conti B.A., Renné T., Strickland S. (2015). Activation of the Factor XII-Driven Contact System in Alzheimer’s Disease Patient and Mouse Model Plasma. Proc. Natl. Acad. Sci. USA.

[114] Mittal M., Siddiqui M.R., Tran K., Reddy S.P., Malik A.B. (2014). Reactive Oxygen Species in Inflammation and Tissue Injury. Antioxid. Redox Signal..

[115] Schieber M., Chandel N.S. (2014). ROS Function in Redox Signaling and Oxidative Stress. Curr. Biol..

[116] Cardoso S., Santos R.X., Correia S.C., Carvalho C., Santos M.S., Baldeiras I., Oliveira C.R., Moreira P.I. (2013). Insulin-Induced Recurrent Hypoglycemia Exacerbates Diabetic Brain Mitochondrial Dysfunction and Oxidative Imbalance. Neurobiol. Dis..

[117] Van de Haar H.J., Burgmans S., Jansen J.F.A., van Osch M.J.P., van Buchem M.A., Muller M., Hofman P.A.M., Verhey F.R.J., Backes W.H. (2016). Blood-Brain Barrier Leakage in Patients with Early Alzheimer Disease. Radiology.

[118] Tkacs N.C., Dunn-Meynell A.A., Levin B.E. (2000). Presumed Apoptosis and Reduced Arcuate Nucleus Neuropeptide Y and pro-Opiomelanocortin mRNA in Non-Coma Hypoglycemia. Diabetes.

[119] Yamada K.A., Rensing N., Izumi Y., De Erausquin G.A., Gazit V., Dorsey D.A., Herrera D.G. (2004). Repetitive Hypoglycemia in Young Rats Impairs Hippocampal Long-Term Potentiation. Pediatr. Res..

[120] He C., Li Q., Cui Y., Gao P., Shu W., Zhou Q., Wang L., Li L., Lu Z., Zhao Y. (2022). Recurrent Moderate Hypoglycemia Accelerates the Progression of Alzheimer’s Disease through Impairment of the TRPC6/GLUT3 Pathway. JCI Insight.

[121] He C., Gao P., Cui Y., Li Q., Li Y., Lu Z., Ma H., Zhao Y., Li L., Sun F. (2020). Low-Glucose-Sensitive TRPC6 Dysfunction Drives Hypoglycemia-Induced Cognitive Impairment in Diabetes. Clin. Transl. Med..

[122] Ishikawa T., Koshizaka M., Maezawa Y., Takemoto M., Tokuyama Y., Saito T., Yokote K. (2018). Continuous Glucose Monitoring Reveals Hypoglycemia Risk in Elderly Patients with Type 2 Diabetes Mellitus. J. Diabetes Investig..

[123] Jackson M.A., Ahmann A., Shah V.N. (2021). Type 2 Diabetes and the Use of Real-Time Continuous Glucose Monitoring. Diabetes Technol. Ther..

[124] Battelino T., Phillip M., Bratina N., Nimri R., Oskarsson P., Bolinder J. (2011). Effect of Continuous Glucose Monitoring on Hypoglycemia in Type 1 Diabetes. Diabetes Care.

[125] Heller S.R., Peyrot M., Oates S.K., Taylor A.D. (2020). Hypoglycemia in Patient with Type 2 Diabetes Treated with Insulin: It Can Happen. BMJ Open Diabetes Res. Care.

[126] Mujahid O., Contreras I., Vehi J. (2021). Machine Learning Techniques for Hypoglycemia Prediction: Trends and Challenges. Sensors.

[127] Shafiee G., Mohajeri-Tehrani M., Pajouhi M., Larijani B. (2012). The Importance of Hypoglycemia in Diabetic Patients. J. Diabetes Metab. Disord..

[128] Dave D., Erraguntla M., Lawley M., DeSalvo D., Haridas B., McKay S., Koh C. (2021). Improved Low-Glucose Predictive Alerts Based on Sustained Hypoglycemia: Model Development and Validation Study. JMIR Diabetes.

[129] Weinstein G., Davis-Plourde K.L., Conner S., Himali J.J., Beiser A.S., Lee A., Rawlings A.M., Sedaghat S., Ding J., Moshier E. (2019). Association of Metformin, Sulfonylurea and Insulin Use with Brain Structure and Function and Risk of Dementia and Alzheimer’s Disease: Pooled Analysis from 5 Cohorts. PLoS ONE.

[130] Tseng C.-H. (2018). Pioglitazone Reduces Dementia Risk in Patients with Type 2 Diabetes Mellitus: A Retrospective Cohort Analysis. J. Clin. Med. Res..

[131] Woldaregay A.Z., Årsand E., Botsis T., Albers D., Mamykina L., Hartvigsen G. (2019). Data-Driven Blood Glucose Pattern Classification and Anomalies Detection: Machine-Learning Applications in Type 1 Diabetes. J. Med. Internet Res..

[132] Mosquera-Lopez C., Dodier R., Tyler N.S., Wilson L.M., El Youssef J., Castle J.R., Jacobs P.G. (2020). Predicting and Preventing Nocturnal Hypoglycemia in Type 1 Diabetes Using Big Data Analytics and Decision Theoretic Analysis. Diabetes Technol. Ther..

[133] Rdzak G.M., Abdelghany O. (2014). Does Insulin Therapy for Type 1 Diabetes Mellitus Protect against Alzheimer’s Disease?. Pharmacotherapy.

[134] Morris J.K., Burns J.M. (2012). Insulin: An Emerging Treatment for Alzheimer’s Disease Dementia?. Curr. Neurol. Neurosci. Rep..

[135] Badenes R., Qeva E., Giordano G., Romero-García N., Bilotta F. (2021). Intranasal Insulin Administration to Prevent Delayed Neurocognitive Recovery and Postoperative Neurocognitive Disorder: A Narrative Review. Int. J. Environ. Res. Public Health.

[136] Long C., Han X., Yang Y., Li T., Zhou Q., Chen Q. (2022). Efficacy of Intranasal Insulin in Improving Cognition in Mild Cognitive Impairment or Dementia: A Systematic Review and Meta-Analysis. Front. Aging Neurosci..

[137] Bhatti G.K., Reddy A.P., Reddy P.H., Bhatti J.S. (2019). Lifestyle Modifications and Nutritional Interventions in Aging-Associated Cognitive Decline and Alzheimer’s Disease. Front. Aging Neurosci..

[138] Choe H.J., Rhee E.-J., Won J.C., Park K.S., Lee W.-Y., Cho Y.M. (2022). Effects of Patient-Driven Lifestyle Modification Using Intermittently Scanned Continuous Glucose Monitoring in Patients With Type 2 Diabetes: Results from the Randomized Open-Label PDF Study. Diabetes Care.

[139] Cholerton B., Baker L.D., Montine T.J., Craft S. (2016). Type 2 Diabetes, Cognition, and Dementia in Older Adults: Toward a Precision Health Approach. Diabetes Spectr..

[140] Martín-Peláez S., Fito M., Castaner O. (2020). Mediterranean Diet Effects on Type 2 Diabetes Prevention, Disease Progression, and Related Mechanisms. A Review. Nutrients.

[141] Abdelbasset W.K., Nambi G., Elsayed S.H., Osailan A.M., Eid M.M. (2021). Falls and Potential Therapeutic Interventions among Elderly and Older Adult Patients with Cancer: A Systematic Review. Afr. Health Sci..

[142] Younk L.M., Mikeladze M., Tate D., Davis S.N. (2011). Exercise-Related Hypoglycemia in Diabetes Mellitus. Expert Rev. Endocrinol. Metab..

[143] Banarer S., Cryer P.E. (2004). Hypoglycemia in Type 2 Diabetes. Med. Clin. N. Am..

[144] Amiel S.A., Dixon T., Mann R., Jameson K. (2008). Hypoglycaemia in Type 2 Diabetes. Diabet. Med..

[145] Wright A.D., Cull C.A., Macleod K.M., Holman R.R. (2006). UKPDS Group Hypoglycemia in Type 2 Diabetic Patients Randomized to and Maintained on Monotherapy with Diet, Sulfonylurea, Metformin, or Insulin for 6 Years from Diagnosis: UKPDS73. J. Diabetes Complicat..

[146] Kahn S.E., Haffner S.M., Heise M.A., Herman W.H., Holman R.R., Jones N.P., Kravitz B.G., Lachin J.M., O’Neill M.C., Zinman B. (2006). Glycemic Durability of Rosiglitazone, Metformin, or Glyburide Monotherapy. N. Engl. J. Med..

[147] Intensive Blood-Glucose Control with Sulphonylureas or Insulin Compared with Conventional Treatment and Risk of Complications in Patients with Type 2 Diabetes (UKPDS 33) (1998). UK Prospective Diabetes Study (UKPDS) Group. Lancet.

[148] Leese G.P., Wang J., Broomhall J., Kelly P., Marsden A., Morrison W., Frier B.M., Morris A.D. (2003). DARTS/MEMO Collaboration Frequency of Severe Hypoglycemia Requiring Emergency Treatment in Type 1 and Type 2 Diabetes: A Population-Based Study of Health Service Resource Use. Diabetes Care.

[149] (2007). UK Hypoglycaemia Study Group Risk of Hypoglycaemia in Types 1 and 2 Diabetes: Effects of Treatment Modalities and Their Duration. Diabetologia.

[150] Stahl M., Berger W. (1999). Higher Incidence of Severe Hypoglycaemia Leading to Hospital Admission in Type 2 Diabetic Patients Treated with Long-Acting versus Short-Acting Sulphonylureas. Diabet. Med..

[151] Clemens K.K., Shariff S., Liu K., Hramiak I., Mahon J.L., McArthur E., Garg A.X. (2015). Trends in Antihyperglycemic Medication Prescriptions and Hypoglycemia in Older Adults: 2002–2013. PLoS ONE.

[152] Brunton S.A. (2012). Hypoglycemic Potential of Current and Emerging Pharmacotherapies in Type 2 Diabetes Mellitus. Postgrad. Med..

[153] Athauda D., Foltynie T. (2016). The Glucagon-like Peptide 1 (GLP) Receptor as a Therapeutic Target in Parkinson’s Disease: Mechanisms of Action. Drug Discov. Today.

[154] Duarte A.I., Candeias E., Correia S.C., Santos R.X., Carvalho C., Cardoso S., Plácido A., Santos M.S., Oliveira C.R., Moreira P.I. (2013). Crosstalk between Diabetes and Brain: Glucagon-like Peptide-1 Mimetics as a Promising Therapy against Neurodegeneration. Biochim. Biophys. Acta.

[155] Patrone C., Eriksson O., Lindholm D. (2014). Diabetes Drugs and Neurological Disorders: New Views and Therapeutic Possibilities. Lancet Diabetes Endocrinol..

[156] Dominguez L.J., Paolisso G., Barbagallo M. (2010). Glucose Control in the Older Patient: From Intensive, to Effective and Safe. Aging Clin. Exp. Res..

[157] Grossman S. (2011). Management of Type 2 Diabetes Mellitus in the Elderly: Role of the Pharmacist in a Multidisciplinary Health Care Team. J. Multidiscip. Healthc..

[158] Suh S.W., Aoyama K., Chen Y., Garnier P., Matsumori Y., Gum E., Liu J., Swanson R.A. (2003). Hypoglycemic Neuronal Death and Cognitive Impairment Are Prevented by poly(ADP-Ribose) Polymerase Inhibitors Administered after Hypoglycemia. J. Neurosci..

[159] Bohio A.A., Sattout A., Wang R., Wang K., Sah R.K., Guo X., Zeng X., Ke Y., Boldogh I., Ba X. (2019). C-Abl-Mediated Tyrosine Phosphorylation of PARP1 Is Crucial for Expression of Proinflammatory Genes. J. Immunol..

[160] Martire S., Fuso A., Rotili D., Tempera I., Giordano C., De Zottis I., Muzi A., Vernole P., Graziani G., Lococo E. (2013). PARP-1 Modulates Amyloid Beta Peptide-Induced Neuronal Damage. PLoS ONE.

[161] Kauppinen T.M., Suh S.W., Higashi Y., Berman A.E., Escartin C., Won S.J., Wang C., Cho S.-H., Gan L., Swanson R.A. (2011). Poly(ADP-Ribose)polymerase-1 Modulates Microglial Responses to Amyloid β. J. Neuroinflamm..

[162] Klonoff D.C. (2008). Personalized Medicine for Diabetes. J. Diabetes Sci. Technol..

[163] Kleinberger J.W., Pollin T.I. (2015). Personalized Medicine in Diabetes Mellitus: Current Opportunities and Future Prospects. Ann. N. Y. Acad. Sci..

